# Supplementary material for: Clinical and genetic associations of deep learning-derived cardiac magnetic resonance-based left ventricular mass
Source: Nat Commun. 2023 Mar 21;14:1558. doi: 10.1038/s41467-023-37173-w (PMC10030590; doi:10.1038/s41467-023-37173-w)
Supplement: Supplementary file 1 — Supplementary Information [file 41467_2023_37173_MOESM1_ESM.pdf]

Clinical and Genetic Associations of Deep Learning-Derived Cardiac Magnetic Resonance-Based Left Ventricular Mass

Supplementary Information

Index

Supplementary Table 1. Baseline characteristics of study samples 1
Supplementary Table 2. Bioinformatics and *in silico* functional analysis summary 2
Supplementary Table 3. Variants with suggestive associations with CMR-derived left ventricular mass index in the mixed-ancestry GWAS 4
Supplementary Table 4. Variants associated with unindexed CMR-derived left ventricular mass 5
Supplementary Table 5. Variants associated with CMR-derived left ventricular mass index in the European-ancestry GWAS 6
Supplementary Table 6. Variants associated with CMR-derived left ventricular mass indexed using the 2.7th power of height 7
Supplementary Table 7. Variants associated with CMR-derived left ventricular mass after rank-based inverse normal transformation 8
Supplementary Table 8. Variants associated with CMR-derived left ventricular mass indexed using lean body mass 9
Supplementary Table 9. Variants associated with CMR-derived left ventricular mass index excluding individuals with heart failure and coronary artery disease............ 10
Supplementary Table 10. Variants associated with unindexed CMR-derived left ventricular mass conditioned on height and weight 11
Supplementary Table 11. Associations with CMR-derived left ventricular mass across varying indexing methods............ 12
Supplementary Table 12. Prior associations of lead variants with CMR-derived left ventricular measurements and cardiovascular traits............ 13
Supplementary Table 13. Associations between cardiac magnetic resonance-derived indexed LVM and incident cardiovascular disease 16
Supplementary Table 14. Associations between LVMI PRS and incident disease in European ancestry subset............ 17
Supplementary Table 15. Associations between LVMI PRS and incident disease using PRS derived among individuals without prevalent myocardial infarction and heart failure 18
Supplementary Table 16. Two-sample Mendelian randomization for LVMI and LVM . 19
Supplementary Table 17. List of HCM and DCM rare variants............ 20

Supplementary Figure 1. Quantile-quantile plot of LVMI and LVM GWAS 24
Supplementary Figure 2. Regional association plots for LVMI genome-wide significant loci 25
Supplementary Figure 3. Manhattan plot of unindexed LV mass GWAS 31
Supplementary Figure 4. Quantile-quantile plot of LVMI GWAS for European ancestry subset 32
Supplementary Figure 5. Manhattan plot of LVMI GWAS in European ancestry subset 33
Supplementary Figure 6. Two-sample Mendelian randomization plots 34
Supplementary Figure 7. Distribution of CMR-derived LV mass 35
Supplementary References. 36

Supplementary Table 1. Baseline characteristics of study samples

	Mean ± SD or N (%)			
	**UKBB Phenotype Sample (N=44,375)***	**UKBB GWAS Sample (N=43,230)***	**UKBB PRS Sample (N=443,326)***	**MGB PRS Sample (N=29,354)***
Age	64.6 ± 7.7	55.6 ± 7.6	57.2 ± 8.1	55.4 ± 16.8
Female	23,061 (52.0%)	22,392 (51.8%)	241,499 (54.5%)	16,008 (54.5%)
Race/ethnicity†	-			
White	42,941 (96.8%)	41,855 (96.8%)	416,675 (94.0%)	24,803 (84.5%)
Asian or Pacific Islander	598 (1.3%)	577 (1.3%)	10,328 (2.3%)	612 (2.1%)
Black	289 (0.7%)	273 (0.6%)	7,359 (1.7%)	1,414 (4.8%)
Other	226 (0.5%)	213 (0.5%)	4,136 (0.9%)	834 (2.8%)
Mixed	204 (0.5%)	198 (0.5%)	2,638 (0.6%)	0 (0%)
Hispanic/Latino	-	-	-	946 (3.2%)
Not reported	117 (0.3%)	114 (0.3%)	2,190 (0.5%)	745 (2.5%)
Hypertension	13,583 (30.6%)	13,225 (30.6%)	130,370 (29.4%)	15,024 (51.2%)
Diabetes	1,752 (3.9%)	1,704 (3.9%)	12,073 (2.7%)	4,727 (16.1%)
Heart failure	317 (0.7%)	306 (0.7%)	2,736 (0.6%)	3,516 (12.0%)
Myocardial infarction	1,110 (2.5%)	1,087 (2.5%)	11,282 (2.5%)	3,445 (11.7%)
CMR-derived LVM index (g/m^2)	47.7 ± 12.2	-	-	-

*Baseline characteristics defined at time of MRI for UKBB Phenotype sample, and time of DNA collection for all other samples
†Self-reported race. MGB demographic data combines race and ethnicity information.

Supplementary Table 2. Bioinformatics and *in silico* functional analysis summary

Mixed-ancestry analysis												
**rsID**	**Chr**	**Position (hg38)**	**Closest gene**	**GTEx v8 eQTL LV***	**GTEx v8 eQTL AA***	**TWAS LV**	**TWAS AA**	**Hi-C linked genes**	**Plausible genes within 500kb**	**Prioritized candidate genes†**	**Distance to lead SNP**	**LV expression level‡**
rs143800963	1	11835418	*CLCN6*	-	*NPPA*	-	-	-	*CLCN6, MTHFR, NPPA, NPPB*	*NPPA*	10291	35.81
										NPPB	22046	26.77
										CLCN6	29322	7.28
rs2255167	2	178693555	*TTN*	*FKBP7*	*FKBP7*	*FKBP7*	*FKBP7*	-	*TTN, FKBP7, CCDC141*	*TTN*	167566	66.76
										FKBP7	229891	2.675
rs10497529	2	178975161	*CCDC141*	-	-	-	-	-	*TTN, CCDC141*	*TTN*	449172	66.76
										CCDC141	145404	5.30
-	5	133066736	*HSPA4*	-	-	-	-	*AFF4*	*HSPA4, ZCCHC10*	*HSPA4*	14723	25.89
rs9388498	6	126552277	*CENPW*	-	-	-	-	-	*CENPW*	*CENPW*	212162	0.42
rs34163229	10	73647154	*SYNPO2L*	*SYNPO2L*	*SYNPO2L*	-	*SYNPO2L*	*USP54*	*MYOZ1, PPP3CB, ANXA7, AGAP5, FUT11, SYNPO2L*	*SYNPO2L*	2273	84.32
				-	*MYOZ1*	-	*MYOZ1*			*MYOZ1*	15542	1.06
				FUT11	*FUT11*	-	-			*ANXA7*	272053	50.75
				-	*AGAP5*	-	*AGAP5*			*AGAP5*	27133	1.74
				-	*DNAJC9*	-	-					
				-	*DUSP8P5*	-	-					
rs3729989	11	47348490	*MYBPC3*	*PSMC3*	*PSMC3*	-	*PSMC3*	-	*MYBPC3, PSMC3*	*MYBPC3*	4467	1,351
										PSMC3	91830	97.09
rs28552516	12	121592356	*KDM2B*	-	MORN3	-	-	*ORAI1, MORN3*	*ORAI1, KDM2B, MORN3*	*ORAI1*	34194	4.91
rs6598541	15	98727906	*IGF1R*	*IGF1R*	-	*IGF1R*	-	-	*IGF1R*	*IGF1R*	79367	5.71
rs56252725	16	14995819	*PDXDC1*	*PDXDC1*	-	*PDXDC1*	-	*MYH11*	*PDXDC1, NOMO1*	*PDXDC1*	21228	15.96
				PKD1P3	-	-	-			*NOMO1*	162098	15.12
				NPIPA3	-	-	-					
				NPIPA5	*NPIPA5*	-	-					
				RRN3	-	-	-					
				-	*NOMO1*	-	-			*MYH11*	801210	16.25
				NPIPA1	*NPIPA1*	-	-					

				-	*AC139256.1*	-	-					
				-	-	*SEZ6L2*	-					
rs6503451	17	45870981	*MAPT*	*MAPT*	-	-	-	-	*MAPT, LRRC37A2, DND1P1, MAPK8IP1P2, KANSL1, ARL17A, WNT3, CRHR1*	*KANSL1*	158935	3.72
				LRRC37A4P	*LRRC37A4P*	-	-			*MAPT*	23546	4.75
				LRRC37A2	*LRRC37A2*	*LRRC37A2*	-			*LRRC37A2*	421752	0.12
				DND1P1	*DND1P1*	-	-					
				MAPK8IP1P2	*MAPK8IP1P2*	-	-					
				LINC02210	*LINC02210*	-	-					
				KANSL1	*KANSL1*	-	-					
				ARL17A	*ARL17A*	-	-					
				WNT3	*WNT3*	-	-					
				-	*NSF*	-	-					
rs199501	17	46784981	*WNT3*	*WNT3*	*WNT3*	*WNT3*	*WNT3*	-	*NSF, WNT3, LRRC37A, LRRC37A2, KANSL1, ARL17A*	*WNT3*	22475	0.41
				LRRC37A	*LRRC37A*	-	-			*KANSL1*	755065	3.72
				LRRC37A2	*LRRC37A2*	-	-			*LRRC37A*	492248	0.12
				ARL17A	*ARL17A*	-	-			*LRRC37A2*	273736	0.12
				NSF	*NSF*	-	-					
				KANSL1	*KANSL1*	-	-					
				MAPT	-	-	-					
rs62621197	19	8605262	*ADAMTS10*	-	-	-	-	*NFILZ*	*ADAMTS10, MYO1F*	*ADAMTS10*	25022	3.79
										MYO1F	84484	1.85
European-ancestry analysis												
rs143973349	7	128866182	*ATP6V1F*	*KCP*	-	*KCP*		*CCDC136, CALU*	*FLNC, ATP6V1F, KCP, CCDC136, OP1SW, IRF5*	*FLNC*	35776	291.60
				-	*CCDC136*	-				*CCDC136*	435371	1.28
										KCP	4140	0.17
rs59765302	16	30018280	*DOC2A*	-	-	-	-	*MVP*	*DOC2A*	*DOC2A*	1450	0.25

*eQTLs determined using lead variants or proxy variants ($r^2 \geq 0.8$) using all-population or European-ancestry linkage disequilibrium maps from the 1,000 Genomes Project
†Candidate genes prioritized on the basis of closest proximity to the lead variant, eQTLs, TWAS, tissue-specific expression levels, Hi-C analysis, and biologic plausibility based on previously reported data and are supported by at least two lines of evidence, except where otherwise specified
‡Expression levels derived from bulk tissue samples available in GTEx v8

Supplementary Table 3. Variants with suggestive associations with CMR-derived left ventricular mass index in the mixed-ancestry GWAS

rsID	Chr	Position (hg 38)	Closest gene(s)	Function	Risk/alt allele	RAF	Beta	SE	P-value*
rs140620427	2	66552028	*MEIS1*	Intronic	G/GAC	0.64	-0.40	0.08	4.8×10^{-7}
rs80197559	3	134708638	*EPHB1*	Intronic	A/T	0.93	0.71	0.14	6.3×10^{-7}
-	3	169759203	*ACTRT3*	-	CAA/C	0.75	0.44	0.09	3.4×10^{-7}
rs13108245	4	56924039	*REST*	Intronic	A/G	0.61	0.38	0.08	7.8×10^{-7}
rs36034102	4	80280894	*FGF5*	Intronic	G/T	0.73	-0.44	0.08	1.0×10^{-7}
rs7708324	5	148540531	*HTR4*	Intronic	A/G	0.62	-0.38	0.08	4.2×10^{-7}
rs234478	6	124040001	*NKAIN2*	Intronic	A/G	0.52	0.39	0.07	1.4×10^{-7}
rs17172722	7	46580714	*IGFBP3*	-	C/T	0.58	-0.40	0.08	1.3×10^{-7}
rs73238147	7		*FLNC*	Intronic	T/C	0.86	0.56	0.11	1.4×10^{-7}
rs35886223	7	129370466	*AHCYL2*	Intronic	G/A	0.67	0.41	0.08	3.1×10^{-7}
rs76540339	10	18538957	*CACNB2*	Intronic	A/C	0.87	0.58	0.11	7.6×10^{-8}
rs12253621	10	70680191	*ADAMTS14*	Intronic	G/C	0.74	0.44	0.09	2.1×10^{-7}
rs111555687	10	89819910	*KIF20B*	-	T/C	0.98	1.30	0.25	1.4×10^{-7}
rs621679	11	1881538	*LSP1*	Intronic	G/A	0.63	-0.41	0.08	6.1×10^{-8}
-	11	47927816	*PTPRJ*	-	CAA../C	0.33	0.40	0.08	9.7×10^{-7}
rs35443	12	115115073	*TBX3*	-	G/C	0.61	0.40	0.08	1.6×10^{-7}
rs73468773	15	65274657	*PARP16*	Intronic	C/G	0.97	-1.02	0.20	6.2×10^{-7}
rs6598541	15	98727906	*IGF1R*	Intronic	A/G	0.36	-0.42	0.08	4.6×10^{-8}

*Denotes two-sided p-value corresponding to BOLT-LMM χ^2 statistic
Chr = chromosome; RAF = risk allele frequency

Supplementary Table 4. Variants associated with unindexed CMR-derived left ventricular mass

rsID	Chr	Position (hg 38)	Closest gene(s)	Function	Risk/alt allele	RAF	Beta	SE	P-value*
rs2255167†	2	178693555	*TTN*	Intronic	T/A	0.81	1.83	0.20	$3.2x10^{-19}$
-	5	133066736	*HSPA4*	Indel	CTT/C	0.72	1.02	0.18	$2.5x10^{-8}$
rs9388498	6	126552277	*CENPW*	-	G/T	0.81	-1.43	0.21	$2.1x10^{-11}$
rs3729989	11	47348490	*MYBPC3*	Missense	T/C	0.87	-1.32	0.24	$3.3x10^{-8}$
rs10878349	12	65933852	*HMGA2*‡	Intronic	A/G	0.49	0.99	0.16	$1.5x10^{-9}$
-	13	49990968	*TRIM13*‡	Indel	ACT/A	0.98	-3.20	0.59	$4.7x10^{-8}$
rs4985155	16	15035602	*PDXDC1*	Intronic	A/G	0.66	1.00	0.17	$5.9x10^{-9}$
rs112555002	16	29909744	*SEZ6L2*‡		T/TTC	0.68	-1.02	0.18	$2.1x10^{-8}$
rs1421085	16	53767042	*FTO*‡	Intronic	T/C	0.60	-1.03	0.17	$7.6x10^{-10}$
rs6503451	17	45870981	*MAPT*	Intronic	T/C	0.67	-1.01	0.18	$1.0x10^{-9}$
rs62621197	19	8605262	*ADAMTS10*	Missense	C/T	0.96	2.85	0.45	$2.8x10^{-10}$

*Denotes two-sided p-value corresponding to BOLT-LMM χ^2 statistic
†Locus previously reported for LV mass[1]
‡Variant association unique to unindexed LV mass
Chr = chromosome; RAF = risk allele frequency

Supplementary Table 5. Variants associated with CMR-derived left ventricular mass index in the European-ancestry GWAS

rsID	Chr	Position (hg 38)	Closest gene(s)	Function	Risk/alt allele	RAF	Beta	SE	P-value*
rs143800963	1	11835418	*CLCN6*	Intronic	C/A	0.95	0.98	0.17	$6.8x10^{-9}$
rs2255167[†]	2	178693555	*TTN*	Intronic	T/A	0.81	0.98	0.10	$5.9x10^{-24}$
rs10497529[‡]	2	178975161	*CCDC141*	Missense	G/A	0.96	1.28	0.21	$1.0x10^{-9}$
-	5	133066736	*HSPA4*	Indel	CTT/C	0.72	0.47	0.09	$4.4x10^{-8}$
rs9388498	6	126552277	*CENPW*	-	G/T	0.81	-0.58	0.10	$5.6x10^{-9}$
rs143973349[§]	7	128866181	*ATP6V1F*	Indel	TGG/T	0.82	0.57	0.10	$1.0x10^{-8}$
rs11000728	10	73644542	*MYOZ1*	Intronic	C/G	0.86	-0.61	0.11	$3.7x10^{-8}$
rs61897389	11	47358536	*SPI1*	Intronic	G/T	0.86	-0.64	0.11	$1.2x10^{-8}$
rs28552516	12	121592356	*KDM2B*	Intronic	C/T	0.85	-0.65	0.11	$1.4x10^{-9}$
rs56252725	16	14995819	*PDXDC1*	Intronic	G/A	0.75	0.53	0.10	$4.5x10^{-8}$
rs142032045[§]	16	30018280	*DOC2A*	Indel	C/CA	0.61	-0.45	0.08	$3.9x10^{-8}$
rs6503451	17	45870981	*MAPT*	Intronic	T/C	0.67	-0.54	0.08	$6.9x10^{-11}$
rs199530[#]	17	46759287	*NSF*	Intronic	G/A	0.24	0.53	0.09	$3.3x10^{-9}$
rs62621197	19	8605262	*ADAMTS10*	Missense	C/T	0.96	1.24	0.21	$3.7x10^{-9}$

*Denotes two-sided p-value corresponding to BOLT-LMM χ^2 statistic
†Locus previously reported for LVM[1]
‡Variant identified in conditional analysis conditioned on lead SNPs (beta, standard error, and p-value are adjusted)
§Variant association unique to European ancestry analysis (excluding variants which are strong proxies [r^2 >0.8] for primary GWAS SNPs)
#Association no longer observed in analysis conditioned on rs6503451
Chr = chromosome; RAF = risk allele frequency

Supplementary Table 6. Variants associated with CMR-derived left ventricular mass indexed using the 2.7th power of height

rsID	Chr	Position (hg 38)	Closest gene(s)	Function	Risk/alt allele	RAF	Beta	SE	P-value*
rs2255167†	2	178693555	*TTN*	Intronic	T/A	0.81	0.41	0.047	1.4×10^{-18}
rs571173399	5	133106107	*HSPA4*	Intronic	T/G	0.77	0.25	0.044	1.1×10^{-8}
rs28552516	12	121592356	*KDM2B*	Intronic	C/T	0.85	-0.30	0.052	4.0×10^{-9}
rs1421085	16	53767042	*FTO*	Intronic	T/C	0.60	-0.25	0.038	4.0×10^{-11}
rs6503451	17	45870981	*MAPT*	Intronic	T/C	0.67	-0.26	0.041	1.3×10^{-10}
rs62071449‡	17	46613342	*NSF*	Intronic	G/A	0.81	-0.29	0.050	7.3×10^{-9}

*Denotes two-sided p-value corresponding to BOLT-LMM χ^2 statistic
†Locus previously reported for LVM[1]
‡Association no longer observed in analysis conditioned on rs6503451
Chr., chromosome; RAF, risk allele frequency

Supplementary Table 7. Variants associated with CMR-derived left ventricular mass after rank-based inverse normal transformation

rsID	Chr	Position (hg 38)	Closest gene(s)	Function	Risk/alt allele	RAF	Beta	SE	P-value*
rs143800963	1	11835418	*CLCN6*	Intronic	C/A	0.95	0.077	0.0133	$6.5x10^{-9}$
rs2255167[†]	2	178693555	*TTN*	Intronic	T/A	0.81	0.079	0.0075	$1.5x10^{-26}$
rs10497529[‡]	2	178975161	*CCDC141*	Missense	G/A	0.96	0.100	0.0165	$6.9x10^{-10}$
rs36034102	4	80280894	*FGF5*	Intronic	G/T	0.73	-0.038	0.0068	$2.7x10^{-8}$
-	5	133066736	*HSPA4*	Indel	CTT/C	0.72	0.041	0.0067	$1.1x10^{-9}$
rs9388498	6	126552277	*CENPW*	-	G/T	0.81	-0.045	0.0079	$3.4x10^{-9}$
rs76540339	10	18538957	*CACNB2*	Intronic	A/C	0.87	0.051	0.0087	$8.2x10^{-9}$
rs34163229	10	73647154	*SYNPO2L*	Missense	G/T	0.86	-0.049	0.0085	$6.6x10^{-9}$
rs3729989	11	47348490	*MYBPC3*	Missense	T/C	0.87	-0.051	0.0087	$8.1x10^{-9}$
rs28552516	12	121592356	*KDM2B*	Intronic	C/T	0.85	-0.050	0.0083	$2.5x10^{-9}$
rs6598541	15	98727906	*IGF1R*	Intronic	A/G	0.36	-0.035	0.0062	$3.9x10^{-8}$
rs11376559	16	14995819	*PDXDC1*	Intronic	C/CA	0.70	0.039	0.0066	$2.4x10^{-9}$
rs6503451	17	45870981	*MAPT*	Intronic	T/C	0.67	-0.043	0.0065	$4.8x10^{-11}$
rs199502[§]	17	46785247	*WNT3*	Intronic	G/A	0.79	-0.044	0.0073	$1.5x10^{-9}$

*Denotes two-sided p-value corresponding to BOLT-LMM χ^2 statistic
†Locus previously reported for LVM[1]
‡Variant identified in conditional analysis conditioned on lead SNPs (beta, standard error, and p-value are adjusted)
§Association no longer observed in analysis conditioned on rs6503451
Chr., chromosome; RAF, risk allele frequency

Supplementary Table 8. Variants associated with CMR-derived left ventricular mass indexed using lean body mass

rsID	Chr	Position (hg 38)	Closest gene(s)	Function	Risk/alt allele	RAF	Beta	SE	P-value*
rs143800963	1	11835418	*CLCN6*	Intronic	C/A	0.95	0.08	0.0149	3.9×10^{-8}
rs4233937	2	66525119	*MEIS1*	Intronic	A/G	0.39	0.04	0.0068	3.8×10^{-8}
rs2255167†	2	178693555	*TTN*	Intronic	T/A	0.81	0.09	0.0084	7.2×10^{-29}
rs10497529‡	2	178975161	*CCDC141*	Missense	G/A	0.96	0.04	0.0069	8.8×10^{-11}
rs36034102	4	80280894	*FGF5*	Intronic	G/T	0.73	-0.04	0.0076	1.5×10^{-8}
-	5	133066736	*HSPA4*	Indel	CTT/C	0.72	0.05	0.0075	4.0×10^{-11}
rs73238147	7	128829863	*FLNC*	Intronic	T/C	0.86	0.05	0.0097	3.8×10^{-8}
rs72814544	10	70695167	*ADAMTS14*	Intronic	G/A	0.77	0.04	0.0079	3.2×10^{-8}
rs28489288	12	121587314	*KDM2B*	Intronic	A/G	0.85	-0.05	0.0093	7.0×10^{-9}
rs6598541	15	98727906	*IGF1R*	Intronic	A/G	0.36	-0.04	0.0070	8.6×10^{-10}
rs5816114	16	15005317	*PDXDC1*	Indel	A/AAAAAAG	0.65	0.04	0.0079	2.4×10^{-8}
rs62073222	17	46297344	*LRRC37A*	Synonymous	A/G	0.27	0.05	0.0082	1.3×10^{-8}
rs62621197	19	8605262	*ADAMTS10*	Missense	C/T	0.96	0.11	0.0185	7.6×10^{-10}

*Denotes two-sided p-value corresponding to BOLT-LMM χ^2 statistic
†Locus previously reported for LVM[1]
‡Variant identified in conditional analysis conditioned on lead SNPs (beta, standard error, and p-value are adjusted)
Chr., chromosome; RAF, risk allele frequency

Supplementary Table 9. Variants associated with CMR-derived indexed left ventricular mass index excluding individuals with heart failure and coronary artery disease

rsID	Chr	Position (hg 38)	Closest gene(s)	Function	Risk/alt allele	RAF	Beta	SE	P-value*
rs143800963	1	11835418	*CLCN6*	Intronic	C/A	0.95	0.08	0.0149	$3.9x10^{-8}$
rs4233937	2	66525119	*MEIS1*	Intronic	A/G	0.39	0.04	0.0068	$3.8x10^{-8}$
rs2255167†	2	178693555	*TTN*	Intronic	T/A	0.81	0.09	0.0084	$7.2x10^{-29}$
rs10497529‡	2	178975161	*CCDC141*	Missense	G/A	0.96	0.12	0.0185	$8.8x10^{-11}$
rs36034102	4	80280894	*FGF5*	Intronic	G/T	0.73	-0.04	0.0076	$1.5x10^{-8}$
-	5	133066736	*HSPA4*	Indel	CTT/C	0.72	0.05	0.0075	$4.0x10^{-11}$
rs73238147	7	128829863	*FLNC*	Intronic	T/C	0.86	0.05	0.0097	$3.8x10^{-8}$
rs72814544	10	70695167	*ADAMTS14*	Intronic	G/A	0.77	0.04	0.0079	$3.2x10^{-8}$
rs28489288	12	121587314	*KDM2B*	Intronic	A/G	0.85	-0.05	0.0093	$7.0x10^{-9}$
rs6598541	15	98727906	*IGF1R*	Intronic	A/G	0.36	-0.04	0.0070	$8.6x10^{-10}$
rs5816114	16	15005317	*PDXDC1*	Indel	A/AAAAAAG	0.65	0.04	0.0079	$2.4x10^{-8}$
rs62073222	17	46297344	*LRRC37A*	Synonymous	A/G	0.27	0.05	0.0082	$1.3x10^{-8}$
rs62621197	19	8605262	*ADAMTS10*	Missense	C/T	0.96	0.11	0.0185	$7.6x10^{-10}$

*Denotes two-sided p-value corresponding to BOLT-LMM χ^2 statistic
†Locus previously reported for LVM[1]
‡Variant identified in conditional analysis conditioned on lead SNPs (beta, standard error, and p-value are adjusted)
Chr., chromosome; RAF, risk allele frequency

Supplementary Table 10. Variants associated with unindexed CMR-derived left ventricular mass conditioned on height and weight

rsID	Chr	Position (hg 38)	Closest gene(s)	Function	Risk/alt allele	RAF	Beta	SE	P-value*
rs143800963	1	11835418	*CLCN6*	Intronic	C/A	0.95	1.64	0.297	$3.3x10^{-8}$
rs2255167†	2	178693555	*TTN*	Intronic	T/A	0.81	1.87	0.168	$3.2x10^{-29}$
-	3	169759203	*ACTRT3*	Indel	CAA/C	0.75	0.85	0.154	$4.5x10^{-8}$
-	5	133066736	*HSPA4*	Indel	CTT/C	0.72	0.85	0.149	$1.0x10^{-8}$
rs62388970	5	148528822	*HTR4*	Intronic	G/A	0.62	-0.77	0.137	$1.7x10^{-8}$
rs13198983	6	126863841	*RSPO3*	-	G/A	0.60	-0.76	0.139	$4.1x10^{-8}$
rs848466	7	77963290	*PHTF2*	-	T/C	0.49	-0.78	0.133	$4.0x10^{-9}$
rs13230127	7	128859806	*ATP6V1F*	Intronic	C/T	0.87	1.13	0.196	$1.1x10^{-8}$
rs35886223	7	129370466	*AHCYL2*	Intronic	G/A	0.67	0.80	0.144	$2.4x10^{-8}$
rs34163229	10	73647154	*SYNPO2L*	Missense	G/T	0.86	-1.21	0.191	$2.0x10^{-10}$
rs111555687	10	89819910	*KIF20B*	-	T/C	0.98	2.54	0.448	$1.4x10^{-8}$
rs4980386	11	1874478	*LSP1*	Intronic	C/A	0.62	-0.83	0.137	$1.5x10^{-9}$
rs35443	12	115115073	*TBX3*	-	G/C	0.61	0.74	0.136	$4.2x10^{-8}$
rs6598541	15	98727906	*IGF1R*	Intronic	A/G	0.36	-0.81	0.139	$5.5x10^{-9}$
rs11376559	16	15037797	*PDXDC1*	Indel	C/CA	0.70	0.83	0.146	$1.2x10^{-8}$
rs77727624	17	45713404	*CRHR1*	Intronic	A/G	0.78	-1.02	0.161	$1.6x10^{-10}$
rs62073222	17	46297344	*LRRC37A*	Synonymous	A/G	0.27	1.12	0.162	$5.9x10^{-12}$
rs151269919	17	46816119	*WNT3*	Indel	A/ACACA…	0.36	0.80	0.144	$3.8x10^{-8}$

*Denotes two-sided p-value corresponding to BOLT-LMM χ^2 statistic
†Locus previously reported for LVM[1]
Chr., chromosome; RAF, risk allele frequency

Supplementary Table 11. Associations with CMR-derived left ventricular mass across varying indexing methods

				**Body surface area**		**Body surface area (rank-inverse normal transformation)**		**2.7th power of height**		**Lean body mass**	
**rsID[†]**	**Chr**	**Position (hg38)**	**Closest gene(s)**	**Beta[†]**	**P-value***	**Beta[‡]**	**P-value***	**Beta[‡]**	**P-value***	**Beta[‡]**	**P-value***
rs143800963	1	11835418	*CLCN6*	0.078	4.2×10^{-9}	0.077	6.5×10^{-9}	0.073	2.5×10^{-7}	0.082	3.9×10^{-8}
rs2255167	2	178693555	*TTN*	0.079	3.2×10^{-26}	0.079	1.5×10^{-26}	0.071	1.4×10^{-18}	0.094	7.2×10^{-29}
rs10497529[§]	2	178975161	*CCDC141*	0.100	3.0×10^{-10}	0.100	6.9×10^{-10}	0.095	6.0×10^{-8}	0.120	8.8×10^{-11}
-	5	133066736	*HSPA4*	0.041	1.6×10^{-9}	0.041	1.1×10^{-9}	0.040	2.7×10^{-8}	0.049	4.0×10^{-11}
rs9388498	6	126552277	*CENPW*	-0.045	4.1×10^{-9}	-0.045	3.4×10^{-9}	-0.042	3.4×10^{-7}	-0.045	2.1×10^{-7}
rs34163229	10	73647154	*SYNPO2L*	-0.049	1.0×10^{-8}	-0.049	6.6×10^{-9}	-0.049	1.1×10^{-7}	-0.051	9.8×10^{-8}
rs3729989	11	47348490	*MYBPC3*	-0.050	1.8×10^{-8}	-0.051	8.1×10^{-9}	-0.047	4.9×10^{-7}	-0.049	8.0×10^{-7}
rs28552516	12	121592356	*KDM2B*	-0.047	1.5×10^{-8}	-0.050	2.5×10^{-9}	-0.052	4.0×10^{-9}	-0.054	7.3×10^{-9}
rs6598541	15	98727906	*IGF1R*	-0.034	4.6×10^{-8}	-0.035	3.9×10^{-8}	-0.035	1.8×10^{-7}	-0.043	1.0×10^{-9}
rs56252725	16	14995819	*PDXDC1*	0.044	3.7×10^{-9}	0.044	3.0×10^{-9}	0.039	9.0×10^{-7}	0.045	8.5×10^{-8}
rs6503451	17	45870981	*MAPT*	-0.042	1.1×10^{-10}	-0.043	4.8×10^{-11}	-0.045	1.3×10^{-10}	-0.041	1.9×10^{-8}
rs199501	17	46785247	*WNT3*	0.043	1.1×10^{9}	0.042	3.1×10^{-9}	0.043	1.0×10^{-8}	0.039	6.1×10^{-7}
rs62621197	19	8605262	*ADAMTS10*	0.091	2.9×10^{-8}	0.088	6.6×10^{-8}	0.060	4.9×10^{-4}	0.110	7.6×10^{-10}

*Denotes two-sided p-value corresponding to BOLT-LMM χ^2 statistic
†Variants shown are those significant in the primary GWAS
‡All betas are presented per 1-standard deviation increase to facilitate comparisons
§Variant identified in conditional analysis conditioned on lead SNPs using respective indexing method (beta, standard error, and p-value are adjusted)

Supplementary Table 12. Prior associations of lead variants with CMR-derived left ventricular measurements and cardiovascular traits

rsID	Chr	Position (hg38)	Prioritized candidate gene(s)	Beta (SE)	P-value*	Prior associations with left ventricular measurements†	Prior associations with cardiovascular traits†
Primary association analysis							
rs143800963	1	11835418	*CLCN6* *NPPA* *NPPB*	0.95 (0.16)	$4.2x10^{-9}$		Systolic BP (rs6669371[2]), NTproBNP levels (rs1023252[3])
rs2255167	2	178693555	*TTN* *FKBP7*	0.97 (0.09)	$3.2x10^{-26}$	LVM (rs225516[1]) LVEDV (rs2042995[1]) LVESV (rs2042995[1], rs2562845[4]) LVESVi (rs2562845[4]) LVEF (rs2042995[1], rs2562845[4])	DCM (rare variants[5]), HCM (rare variants[6]), atrial fibrillation (rare variants[7])
rs10497529	2	178975161	*TTN* *CCDC141*	1.28 (0.20)	$2.2x10^{-9}$		Resting heart rate (rs10497529[8]), peak oxygen consumption (rs10497529[9])
-	5	133066736	*HSPA4*	0.50 (0.08)	$1.6x10^{-9}$		Systolic BP (rs62374461[10]), diastolic BP (rs55747751[11])
rs9388498	6	126552277	*CENPW*	-0.55 (0.10)	$4.1x10^{-9}$		Coronary artery disease (rs1591805[12]), Type 1 diabetes (rs1538171[13]), Type 2 diabetes (rs4897182[14])
rs34163229	10	73647154	*SYNPO2L* *MYOZ1* *ANXA7* *PPP3CB*	-0.60 (0.10)	$1.0x10^{-8}$		Systolic BP (rs12247028[15]), atrial fibrillation (multiple[16]), heart failure

			AGAP5				(rs4746140[17]), QT interval (rs4746140[18]), PR interval (rs7394152[19])
rs3729989	11	47348490	*MYBPC3* *PSMC3*	-0.61 (0.11)	$1.8x10^{-8}$		Systolic BP (rs2301216[10]), renin-angiotensin system inhibitor use (rs2856653[20]), HCM (rare variants[21]), DCM (rare variants[22])
rs28552516	12	121592356	*ORAI1*	-0.58 (0.10)	$1.5x10^{-8}$		
rs6598541	15	98727906	*IGF1R*	-0.42 (0.08)	$4.6x10^{-8}$		TPE interval (rs2871974[23]), QRS duration (rs4966020[24]), atrial fibrillation (rs4965430[25])
rs56252725	16	14995819	*PDXDC1* *NOMO1* *MYH11*	0.54 (0.09)	$3.7x10^{-9}$		Coronary artery disease (rs216158[26]), PR interval (rs72772025[19]), resting heart rate (rs3915499[27]), systolic BP (rs3915499[10]), ECG morphology (rs3915425[28])
rs6503451	17	45870981	*KANSL1* *MAPT* *LRRC37A* *LRRC37A2*	-0.52 (0.08)	$1.1x10^{-10}$	LVESV (rs242562[1]) LVESVi (rs242562[1])	Systolic BP (rs3785880[10]), QRS amplitude (rs242562)[29]
rs199501	17	46785247	*WNT3* *KANSL1* *LRRC37A* *LRRC37A2*	0.55 (0.09)	$1.1x10^{9}$	LVESV (rs242562[1]) LVESVi (rs242562[1])	Atrial fibrillation (rs1563304[25])

rs62621197	19	8605262	*ADAMTS10*<br>*MYO1F*	1.11<br>(0.20)	$2.9x10^{-8}$		
European ancestry analysis							
rs143973349	7	128866181	FLNC<br>CCDC136<br>KCP	0.57<br>(0.10)	$1.0x10^{-08}$	LVESV (rs34373805[1])<br>LVESVi (rs34373805[1])<br>LVEF (rs3807309[1])	ECG morphology (rs56216811[28]), arrhythmogenic CM (rare variants[30]), HCM (rare variants[31]), restrictive CM (rare variants[32])
rs142032045	16	30018280	*DOC2A*	-0.45<br>(0.08)	$3.9x10^{-08}$		

*Denotes two-sided p-value corresponding to BOLT-LMM χ^2 statistic
†Traits listed for prior associations with the respective lead variant, a strong proxy ($r^2 \geq 0.80$), or a variant mapped to the same gene. The variant implicated in the prior analysis is listed in parenthesis.
BP = blood pressure, Chr = chromosome; CM = cardiomyopathy, DCM = dilated cardiomyopathy, ECG = electrocardiogram, HCM = hypertrophic cardiomyopathy, LVEF = left ventricular ejection fraction; LVESV = left ventricular end-systolic volume; LVESVi = left ventricular end-systolic volume index, SE = standard error, TPE = T wave peak-to-end

Supplementary Table 13. Associations between cardiac magnetic resonance-derived indexed LVM and incident cardiovascular disease

			Hazard ratio for covariate (95% CI)†		
	**N events / N total***	**Follow-up, years (Q1,Q3)**	**LVMI (per 1 SD)**	**LVH**	**LVH (90th percentile)**
Atrial fibrillation	559 / 43004	2.7 (1.9, 4.1)	1.46 (1.36-1.58)	2.53 (2.02-3.17)	2.37 (1.92-2.92)
Myocardial infarction	299 / 43217	2.7 (1.9, 4.1)	1.32 (1.19-1.47)	1.57 (1.09-2.26)	1.53 (1.10-2.13)
Heart failure	267 / 44011	2.7 (1.9, 4.1)	1.89 (1.72-2.07)	4.95 (3.77-6.50)	4.10 (3.15-5.35)
Ventricular arrhythmias	110 / 44194	2.7 (1.9, 4.1)	1.53 (1.30-1.79)	2.83 (1.74-4.60)	2.35 (1.47-3.75)
Dilated cardiomyopathy	22 / 44297	2.7 (1.9, 4.1)	2.75 (2.20-3.44)	10.8 (4.67-25.2)	11.4 (4.91-26.4)
Hypertrophic cardiomyopathy	14 / 44295	2.7 (1.9, 4.1)	2.39 (1.83-3.12)	7.58 (2.46-23.3)	5.97 (1.86-17.4)
Implantable defibrillator	26 / 44327	2.7 (1.9, 4.1)	2.43 (1.95-3.03)	8.42 (3.81-18.6)	9.53 (4.41-20.6)

*Hazard ratios obtained using Cox proportional hazards models adjusted for age and sex
†N includes all individuals without the prevalent condition at the time of CMR acquisition
CI = confidence interval, LVMI = left ventricular mass index, LVH = left ventricular hypertrophy, Q1 = quartile 1, Q3 = quartile 3, SD = standard deviation

Supplementary Table 14. Associations between LVMI PRS and incident disease in European ancestry subset

			Hazard ratio for covariate (95% CI)*		
**Disease**	**N events / N total**†	**Follow-up, yrs (Q1,Q3)**	**PRS (per 1 SD)**	**PRS (90th percentile)**	**PRS (95th percentile)**
UK Biobank					
Atrial fibrillation	22924 / 380879	11.8 (11.0, 12.6)	1.01 (1.00-1.03)	1.04 (0.99-1.08)	1.05 (0.99-1.12)
Myocardial infarction	11773 / 377679	11.8 (11.0, 12.6)	1.03 (1.01-1.05)	1.07 (1.00-1.14)	1.10 (1.02-1.20)
Heart failure	12080 / 385221	11.8 (11.0, 12.6)	1.05 (1.03-1.07)	1.09 (1.03-1.15)	1.12 (1.04-1.21)
Ventricular arrhythmias	4316 / 386754	11.8 (11.0, 12.6)	1.07 (1.04-1.10)	1.22 (1.11-1.34)	1.34 (1.19-1.52)
Dilated cardiomyopathy	904 / 387385	11.8 (11.0, 12.6)	1.12 (1.05-1.20)	1.16 (0.96-1.43)	1.45 (1.12-1.87)
Hypertrophic cardiomyopathy	327 / 387524	11.8 (11.0, 12.6)	1.18 (1.06-1.32)	1.41 (1.03-1.94)	1.79 (1.22-2.64)
Implantable defibrillator	1175 / 387574	11.8 (11.0, 12.6)	1.08 (1.02-1.14)	1.24 (1.05-1.47)	1.18 (0.93-1.49)
Mass General Brigham					
Atrial fibrillation	1189 / 21096	2.9 (2.0, 4.1)	1.04 (0.98-1.10)	1.09 (0.90-1.31)	1.11 (0.86-1.43)
Myocardial infarction	593 / 21525	3.0 (2.0, 4.1)	0.99 (0.91-1.08)	1.05 (0.81-1.37)	1.10 (0.77-1.57)
Heart failure	940 / 21100	2.9 (2.0, 4.1)	0.99 (0.92-1.05)	0.98 (0.79-1.22)	1.02 (0.76-1.37)
Ventricular arrhythmias	839 / 22684	3.0 (2.0, 4.2)	0.97 (0.91-1.04)	1.11 (0.89-1.38)	0.98 (0.72-1.35)
Dilated cardiomyopathy	401 / 2326	3.0 (2.1, 4.3)	1.09 (0.99-1.20)	1.17 (0.86-1.59)	1.02 (0.66-1.59)
Hypertrophic cardiomyopathy	147 / 24240	3.0 (2.1, 4.3)	1.12 (0.95-1.32)	1.20 (0.72-1.99)	1.24 (0.63-2.45)
Implantable defibrillator	125 / 24000	3.0 (2.1, 4.3)	0.92 (0.77-1.09)	0.93 (0.50-1.72)	1.01 (0.45-2.30)

*Hazard ratios obtained using Cox proportional hazards models adjusted for age, sex, and PCs 1-5
†N includes all individuals without the prevalent condition at baseline

Supplementary Table 15. Associations between LVMI PRS and incident disease using PRS derived among individuals without prevalent myocardial infarction and heart failure

			Hazard ratio for covariate (95% CI)*		
**Disease**	**N events / N total†**	**Follow-up, yrs (Q1, Q3)**	**PRS (per 1 SD)**	**PRS (90th percentile)**	**PRS (95th percentile)**
UK Biobank					
Atrial fibrillation	25050 / 435917	11.8 (11.0, 12.6)	1.02 (1.01-1.03)	1.03 (0.99-1.07)	1.06 (1.00-1.12)
Myocardial infarction	13405 / 432044	11.8 (11.0, 12.6)	1.02 (1.00-1.04)	1.04 (0.98-1.10)	1.03 (0.96-1.12)
Heart failure	13540 / 440590	11.9 (11.0, 12.6)	1.03 (1.02-1.05)	1.08 (1.02-1.14)	1.13 (1.05-1.22)
Ventricular arrhythmias	4882 / 442295	11.9 (11.1, 12.6)	1.05 (1.02-1.08)	1.12 (1.02-1.22)	1.13 (1.00-1.28)
Dilated cardiomyopathy	1023 / 443013	11.9 (11.1, 12.6)	1.08 (1.02-1.15)	1.10 (0.90-1.34)	1.29 (1.00-1.65)
Hypertrophic cardiomyopathy	420 / 443150	11.9 (11.1, 12.6)	1.11 (1.00-1.22)	1.02 (0.74-1.40)	1.29 (0.87-0.92)
Implantable defibrillator	1444 / 443216	11.9 (11.1, 12.6)	1.07 (1.02-1.13)	1.17 (1.00-1.38)	1.37 (1.11-1.68)
Mass General Brigham					
Atrial fibrillation	1332 / 25316	2.9 (2.0, 4.1)	1.02 (0.97-1.08)	1.12 (0.95-1.33)	1.13 (0.89-1.42)
Myocardial infarction	695 / 25592	2.9 (2.0, 4.1)	0.96 (0.89-1.03)	0.87 (0.67-1.13)	0.81 (0.56-1.17)
Heart failure	1074 / 25063	2.9 (2.0, 4.1)	0.96 (0.90-1.02)	1.01 (0.83-1.23)	0.96 (0.73-1.26)
Ventricular arrhythmias	944 / 26990	3.0 (2.0, 4.2)	0.96 (0.90-1.02)	0.94 (0.75-1.16)	0.85 (0.62-1.16)
Dilated cardiomyopathy	492 / 28821	3.0 (2.1, 4.2)	1.08 (0.98-1.18)	1.09 (0.82-1.45)	1.17 (0.80-1.71)
Hypertrophic cardiomyopathy	183 / 28731	3.0 (2.1, 4.2)	1.12 (0.96-1.29)	1.13 (0.71-1.80)	0.90 (0.44-1.83)
Implantable defibrillator	152 / 28454	3.0 (2.1, 4.2)	1.06 (0.90-1.24)	1.40 (0.87-2.25)	2.20 (1.29-3.76)

*Hazard ratios obtained using Cox proportional hazards models adjusted for age, sex, and PCs 1-5
†N includes all individuals without the prevalent condition at baseline

Supplementary Table 16. Two-sample Mendelian randomization for LVMI and LVM

Inverse-variance weighted

Phenotype	LVMI Beta (95% CI)*	LVMI p†	LVM Beta (95% CI)*	LVM p†
Systolic blood pressure	0.27 (0.23-0.31)	1.75x10^{-41}	0.44 (0.35-0.53)	8.16x10^{-23}
Diastolic blood pressure	0.32 (0.25-0.39)	1.64x10^{-20}	0.54 (0.39-0.69)	1.74x10^{-12}
Diabetes	0.31 (0.05-0.56)	0.018	0.62 (0.004-1.23)	0.048

Weighted median

Phenotype	LVMI Beta (95% CI)*	LVMI p†	LVM Beta (95% CI)*	LVM p†
Systolic blood pressure	0.28 (0.23-0.33)	6.65x10^{-28}	0.49 (0.37-0.60)	1.93x10^{-17}
Diastolic blood pressure	0.33 (0.25-0.42)	3.03x10^{-14}	0.59 (0.40-0.78)	7.82x10^{-10}
Diabetes	0.19 (-0.15-0.53)	0.26	0.02 (-0.73-0.77)	0.96

MR-Egger

Phenotype	LVMI Beta (95% CI)*	LVMI p†	LVMI Intercept (p-value)‡	LVM Beta (95% CI)*	LVM p†	LVM Intercept (p-value)‡
Systolic blood pressure	0.24 (0.16-0.31)	3.23x10^{-10}	0.01 (0.38)	0.38 (0.21-0.55)	1.07x10^{-5}	0.02 (0.40)
Diastolic blood pressure	0.42 (0.30-0.54)	1.56x10^{-12}	-0.02 (0.04)	0.76 (0.51-1.02)	6.87x10^{-9}	-0.03 (0.04)
Diabetes	0.15 (-0.36-0.66)	0.56	0.01 (0.50)	-0.33 (-1.56-0.89)	0.59	0.06 (0.08)

*Beta estimates represent the expected causal effect per 1-standard deviation increase in the respective risk factor (phenotype) on LVMI and LVM, respectively
†Denotes two-sided p-value corresponding to the beta estimate from the respective regression model
‡A non-zero intercept suggests the presence of directional pleiotropy, where the two-sided p-value <0.05 indicates a statistically significant difference from zero

Supplementary Table 17. List of HCM and DCM rare variants

Chr	Position (hg 38)	Reference Allele	Alternative Allele	Disease	Class	Gene
1	201365291	C	A	HCM	pathogenic/likely pathogenic	TNNT2
1	236731315	G	A	HCM	loss-of-function	ACTN2
1	53210561	G	A	HCM	missense	CPT2
1	53210561	G	A	HCM	pathogenic/likely pathogenic	CPT2
1	99916398	A	G	HCM	pathogenic/likely pathogenic	AGL
2	219418497	C	T	HCM	pathogenic/likely pathogenic	DES
6	118558982	C	CCT	HCM	loss-of-function	PLN
7	128852972	G	A	HCM	missense	FLNC
11	47332832	C	CG	HCM	loss-of-function	MYBPC3
11	47335081	CAG	C	HCM	loss-of-function	MYBPC3
11	47335081	CAG	C	HCM	pathogenic/likely pathogenic	MYBPC3
11	47337729	A	AC	HCM	pathogenic/likely pathogenic	MYBPC3
11	47337729	A	AC	HCM	loss-of-function	MYBPC3
11	47339375	TG	T	HCM	loss-of-function	MYBPC3
11	47339375	TG	T	HCM	pathogenic/likely pathogenic	MYBPC3
11	47342574	T	A	HCM	pathogenic/likely pathogenic	MYBPC3
11	47343069	G	A	HCM	loss-of-function	MYBPC3
11	47343069	G	A	HCM	pathogenic/likely pathogenic	MYBPC3
11	47343314	C	T	HCM	pathogenic/likely pathogenic	MYBPC3
11	47346379	C	T	HCM	pathogenic/likely pathogenic	MYBPC3
11	47348541	C	G	HCM	pathogenic/likely pathogenic	MYBPC3
11	47352622	C	T	HCM	loss-of-function	MYBPC3
14	23415651	C	T	HCM	pathogenic/likely pathogenic	MYH7
14	23422267	C	T	HCM	missense	MYH7
14	23422267	C	T	HCM	pathogenic/likely pathogenic	MYH7
14	23424107	G	C	HCM	pathogenic/likely pathogenic	MYH7
14	23424839	C	T	HCM	pathogenic/likely pathogenic	MYH7
14	23424909	T	C	HCM	pathogenic/likely pathogenic	MYH7
14	23429850	C	T	HCM	pathogenic/likely pathogenic	MYH7
15	84840481	AG	A	HCM	loss-of-function	ALPK3
15	84862655	AC	A	HCM	loss-of-function	ALPK3
15	84862795	C	G	HCM	loss-of-function	ALPK3
17	80117016	G	C	HCM	pathogenic/likely pathogenic	GAA
18	31592974	G	A	HCM	pathogenic/likely pathogenic	TTR

19	55154095	G	A	HCM	pathogenic/likely pathogenic	TNNI3
1	74489223	T	TA	DCM	loss-of-function	TNNI3K
2	178532133	C	A	DCM	loss-of-function	TTN
2	178532844	G	A	DCM	pathogenic/likely pathogenic	TTN
2	178532844	G	A	DCM	loss-of-function	TTN
2	178533652	G	GTTGA	DCM	loss-of-function	TTN
2	178537725	ATT	A	DCM	loss-of-function	TTN
2	178539559	G	A	DCM	pathogenic/likely pathogenic	TTN
2	178539559	G	A	DCM	loss-of-function	TTN
2	178542492	C	A	DCM	loss-of-function	TTN
2	178543853	T	TG	DCM	loss-of-function	TTN
2	178545817	TACTG	T	DCM	pathogenic/likely pathogenic	TTN
2	178545817	TACTG	T	DCM	loss-of-function	TTN
2	178552221	T	A	DCM	loss-of-function	TTN
2	178552996	ATAAT	A	DCM	pathogenic/likely pathogenic	TTN
2	178552996	ATAAT	A	DCM	loss-of-function	TTN
2	178553039	C	T	DCM	loss-of-function	TTN
2	178553039	C	T	DCM	pathogenic/likely pathogenic	TTN
2	178554149	C	T	DCM	loss-of-function	TTN
2	178554619	TG	T	DCM	loss-of-function	TTN
2	178559385	TAATA	T	DCM	loss-of-function	TTN
2	178562716	G	A	DCM	loss-of-function	TTN
2	178562716	G	A	DCM	pathogenic/likely pathogenic	TTN
2	178562889	A	C	DCM	loss-of-function	TTN
2	178569478	G	A	DCM	pathogenic/likely pathogenic	TTN
2	178569478	G	A	DCM	loss-of-function	TTN
2	178572193	G	A	DCM	loss-of-function	TTN
2	178582434	CAG	C	DCM	loss-of-function	TTN
2	178584726	G	A	DCM	loss-of-function	TTN
2	178584726	G	A	DCM	pathogenic/likely pathogenic	TTN
2	178592401	GAC	G	DCM	loss-of-function	TTN
2	178592602	AC	A	DCM	loss-of-function	TTN
2	178592602	AC	A	DCM	pathogenic/likely pathogenic	TTN
2	178592916	CAG	C	DCM	pathogenic/likely pathogenic	TTN
2	178592916	CAG	C	DCM	loss-of-function	TTN
2	178599242	CT	C	DCM	loss-of-function	TTN
2	178609960	CTACA	C	DCM	pathogenic/likely pathogenic	TTN

2	178609960	CTACA	C	DCM	loss-of-function	TTN
2	178613006	C	T	DCM	loss-of-function	TTN
2	178619714	G	A	DCM	loss-of-function	TTN
2	178621474	C	A	DCM	loss-of-function	TTN
2	178630923	G	A	DCM	loss-of-function	TTN
2	178680253	C	T	DCM	loss-of-function	TTN
2	178689897	C	T	DCM	loss-of-function	TTN
2	178758982	A	G	DCM	loss-of-function	TTN
2	178759017	G	A	DCM	loss-of-function	TTN
2	178799623	T	A	DCM	loss-of-function	TTN
2	219418497	C	T	DCM	pathogenic/likely pathogenic	DES
2	73454058	C	T	DCM	pathogenic/likely pathogenic	ALMS1
2	73572648	AC	A	DCM	loss-of-function	ALMS1
2	73572648	AC	A	DCM	pathogenic/likely pathogenic	ALMS1
2	73573323	C	T	DCM	pathogenic/likely pathogenic	ALMS1
2	73573323	C	T	DCM	loss-of-function	ALMS1
3	136301086	A	G	DCM	missense	PCCB
3	136327173	G	GTA	DCM	loss-of-function	PCCB
3	136327175	G	A	DCM	missense	PCCB
3	136327178	AT	A	DCM	loss-of-function	PCCB
3	136327178	AT	A	DCM	pathogenic/likely pathogenic	PCCB
3	38566533	A	G	DCM	missense	SCN5A
3	38586037	C	T	DCM	missense	SCN5A
5	240459	C	T	DCM	loss-of-function	SDHA
5	240459	C	T	DCM	pathogenic/likely pathogenic	SDHA
6	7579995	C	T	DCM	loss-of-function	DSP
6	7579995	C	T	DCM	pathogenic/likely pathogenic	DSP
6	7580118	A	T	DCM	loss-of-function	DSP
6	7580609	CA	C	DCM	loss-of-function	DSP
6	7580609	CA	C	DCM	pathogenic/likely pathogenic	DSP
6	7580872	ACT	A	DCM	loss-of-function	DSP
6	7584825	TAGAA	T	DCM	pathogenic/likely pathogenic	DSP
6	7584825	TAGAA	T	DCM	loss-of-function	DSP
6	7585228	TGCACA	T	DCM	loss-of-function	DSP
9	105635257	T	TA	DCM	loss-of-function	FKTN
10	110780967	CT	C	DCM	loss-of-function	RBM20
10	110831058	CA	C	DCM	loss-of-function	RBM20

10	119676479	C	T	DCM	loss-of-function	BAG3
10	119676479	C	T	DCM	pathogenic/likely pathogenic	BAG3
10	74118141	G	A	DCM	loss-of-function	VCL
11	19182747	CTG	C	DCM	loss-of-function	CSRP3
12	21805251	C	CA	DCM	loss-of-function	ABCC9
12	21805253	A	T	DCM	loss-of-function	ABCC9
12	21805308	G	A	DCM	missense	ABCC9
12	32802499	GGGTGT	G	DCM	loss-of-function	PKP2
14	23422178	A	C	DCM	loss-of-function	MYH7
Variants listed comprise high confidence loss-of-function, deleterious missense, and known pathogenic or likely pathogenic variants in HCM and DCM genes as catalogued in ClinVar as of 2/9/2021 (see **Supplementary Methods**)						

Supplementary Figure 1. Quantile-quantile plot of LVMI and LVM GWAS

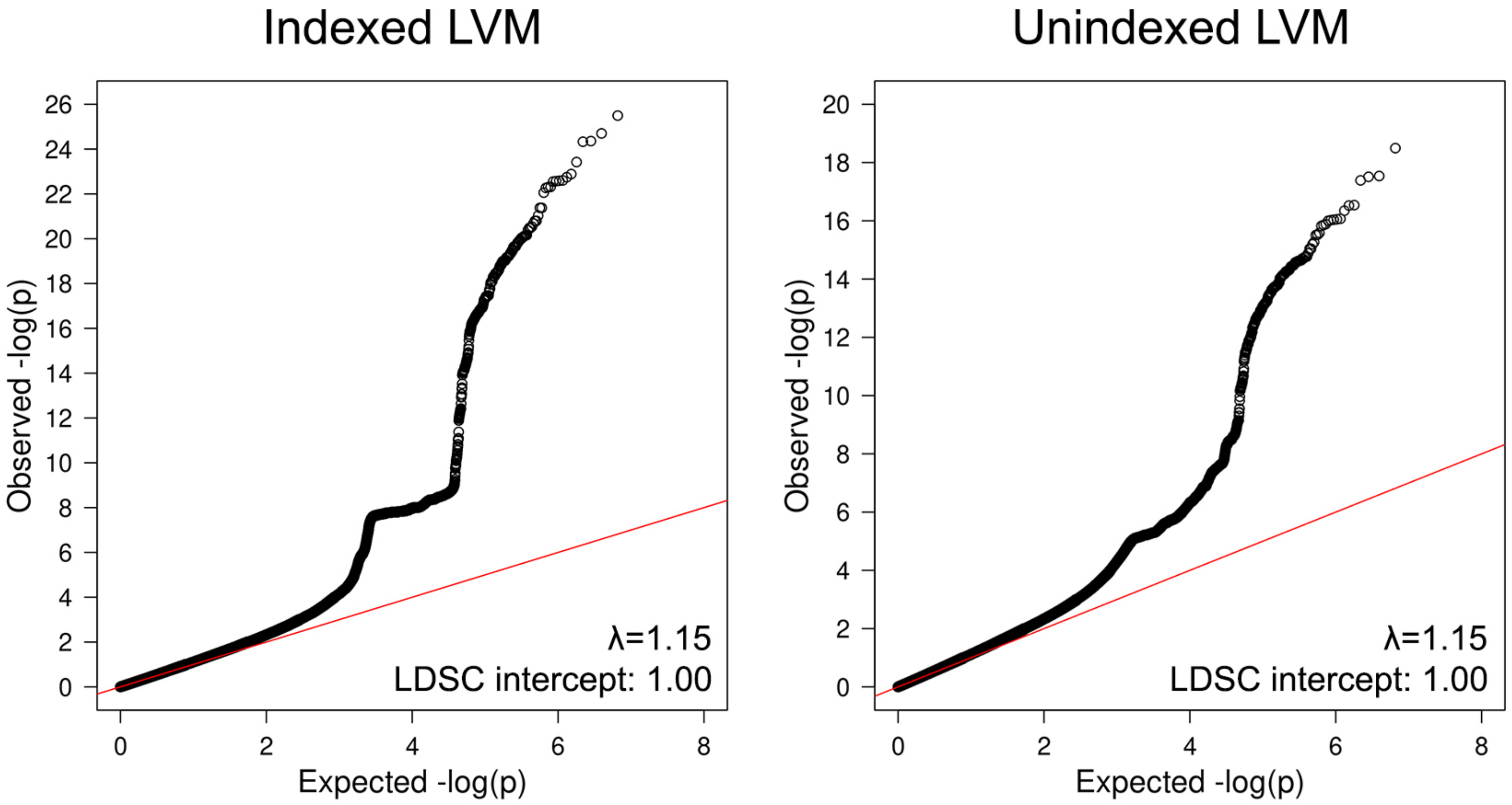

Depicted are quantile-quantile plots for the primary mixed-ancestry LVMI GWAS (left) and the unindexed LVM GWAS (right). The genomic control factor λ is depicted on the bottom right of each plot, as is the linkage disequilibrium score regression intercept, where a value close of one suggests the absence of inflation (and where an elevated λ is instead attributable to polygenicity).[33] Points denote two-sided p-values corresponding to the BOLT-LMM χ^2 statistic.

Supplementary Figure 2. Regional association plots for genome-wide significant loci

rs143800963

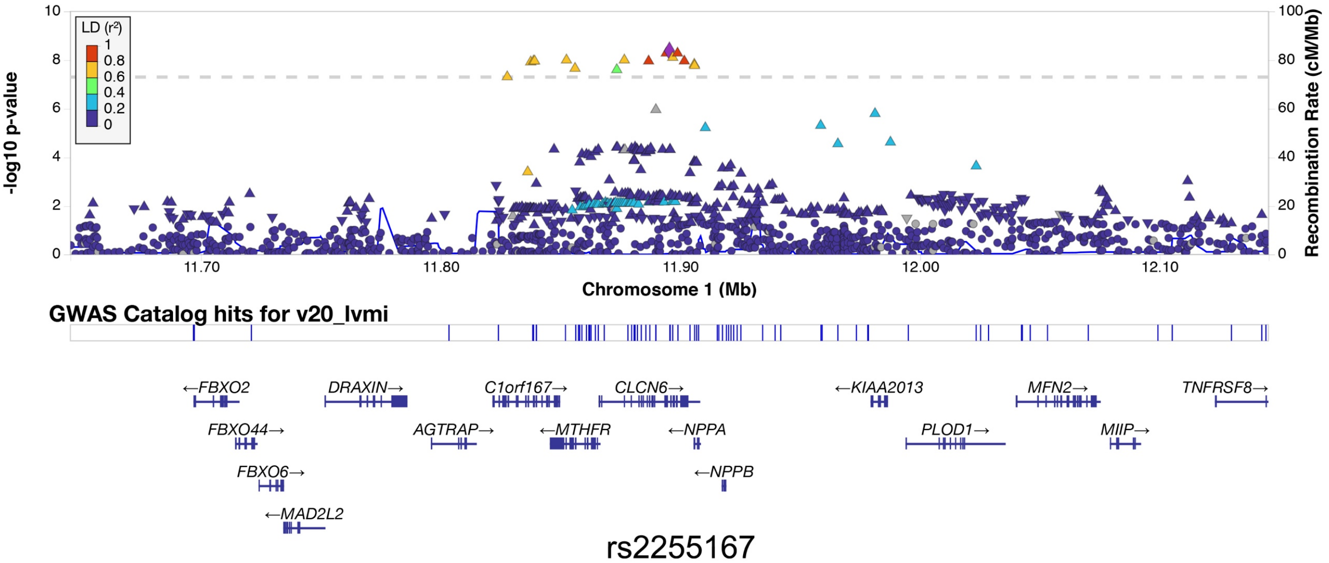

rs2255167

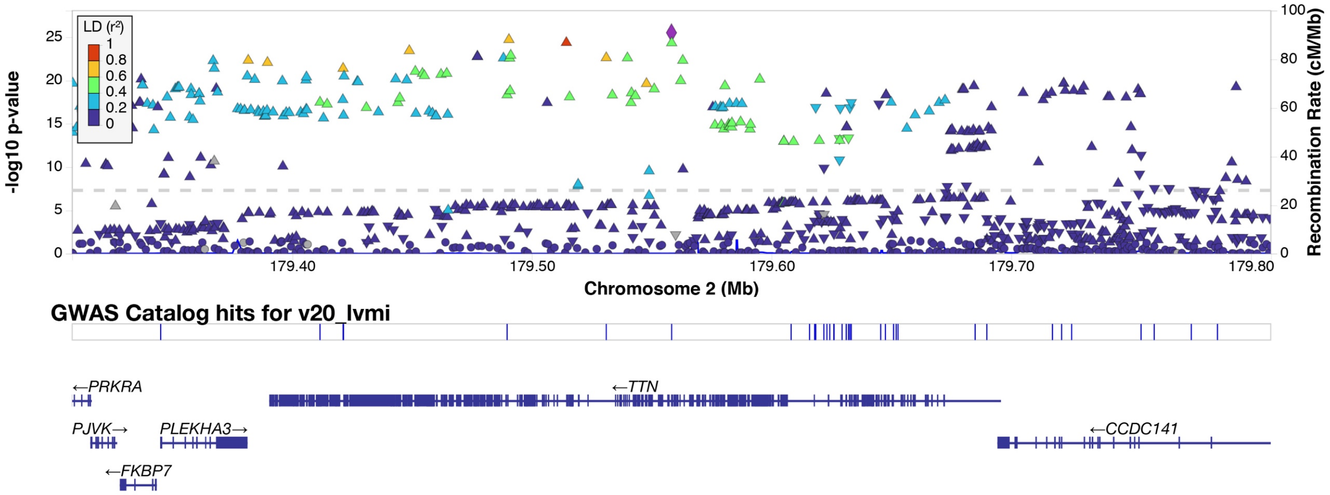

rs10497529

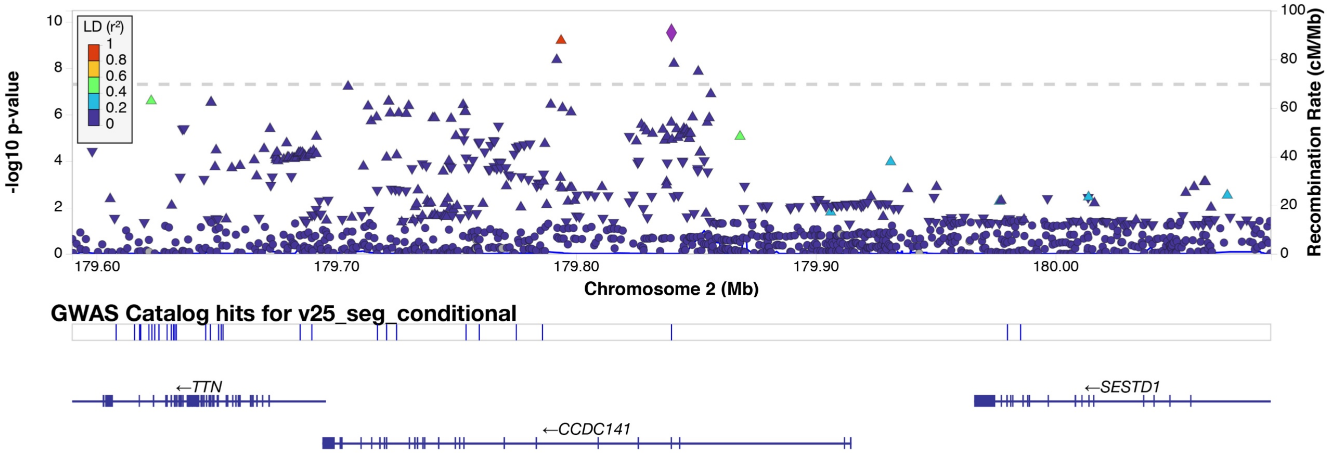

132402428:CTT_C

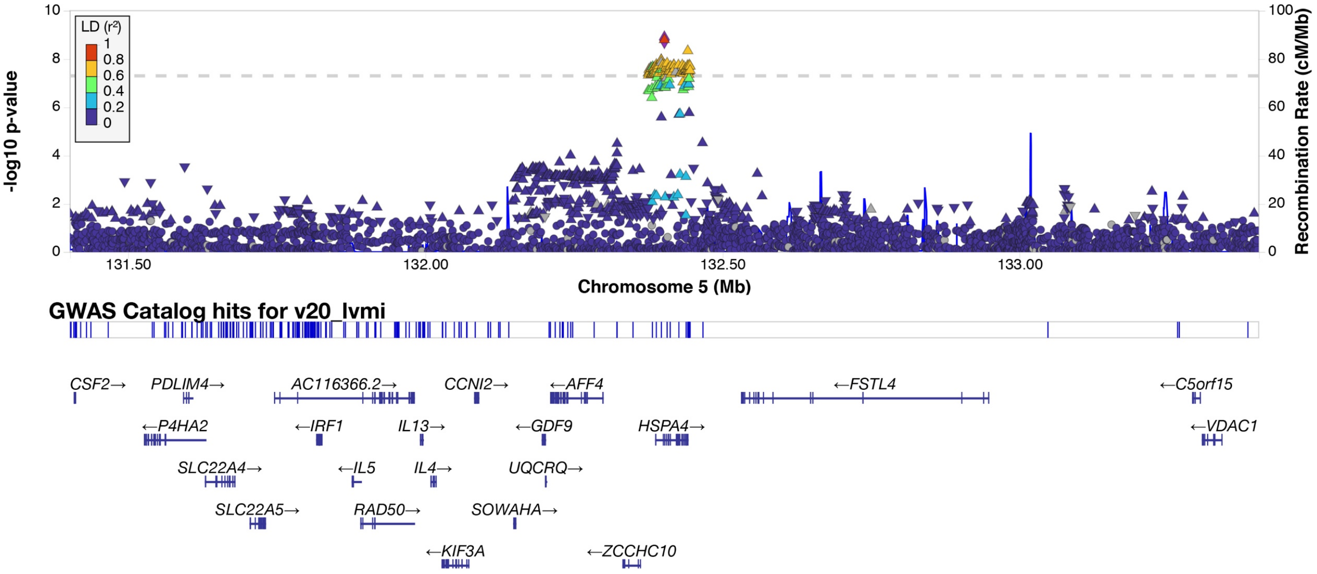

rs9388498

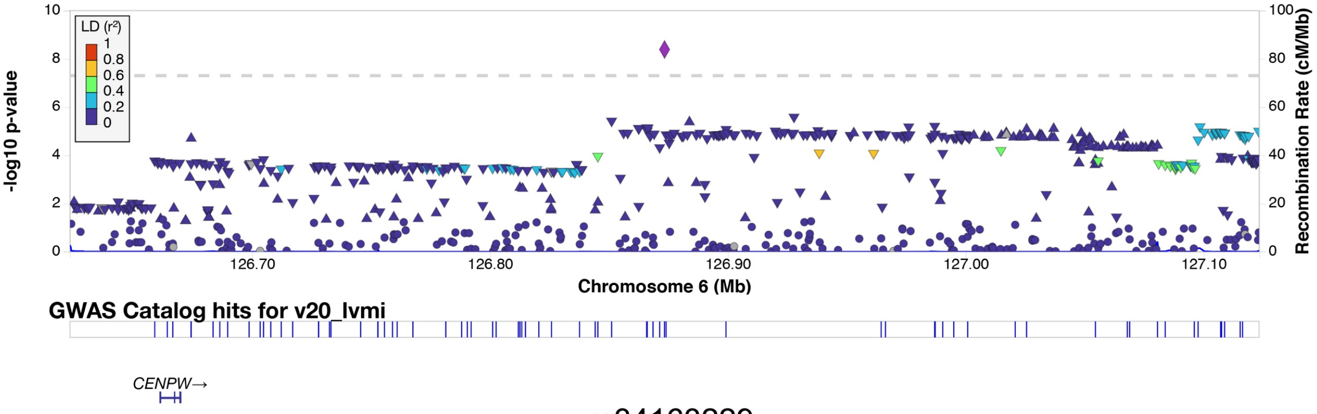

rs34163229

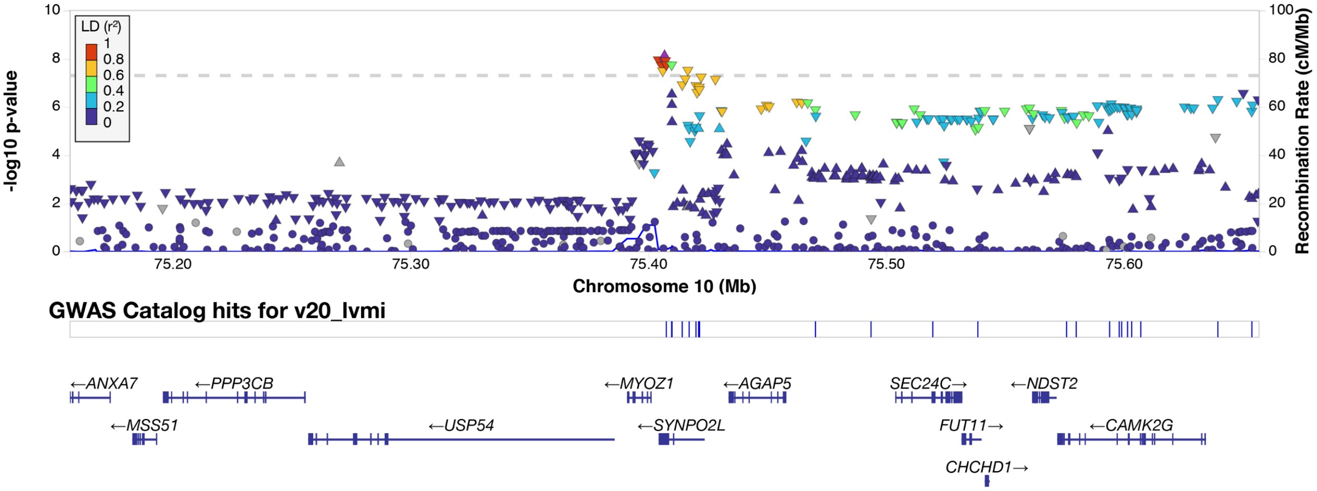

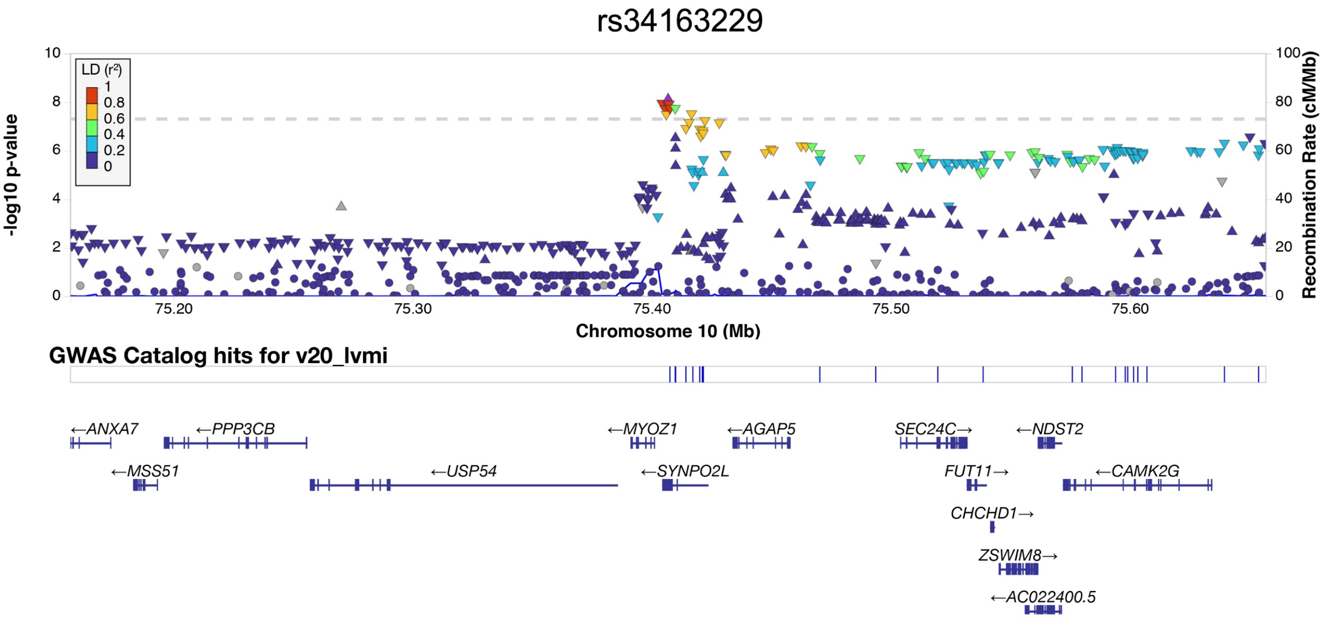
rs34163229
-log10 p-value
Recombination Rate (cM/Mb)
LD (r²)
Chromosome 10 (Mb)
GWAS Catalog hits for v20_lvmi
←ANXA7
←PPP3CB
←MSS51
←USP54
←MYOZ1
←SYNPO2L
←AGAP5
SEC24C→
←NDST2
FUT11→
←CAMK2G
CHCHD1→
ZSWIM8→
←AC022400.5

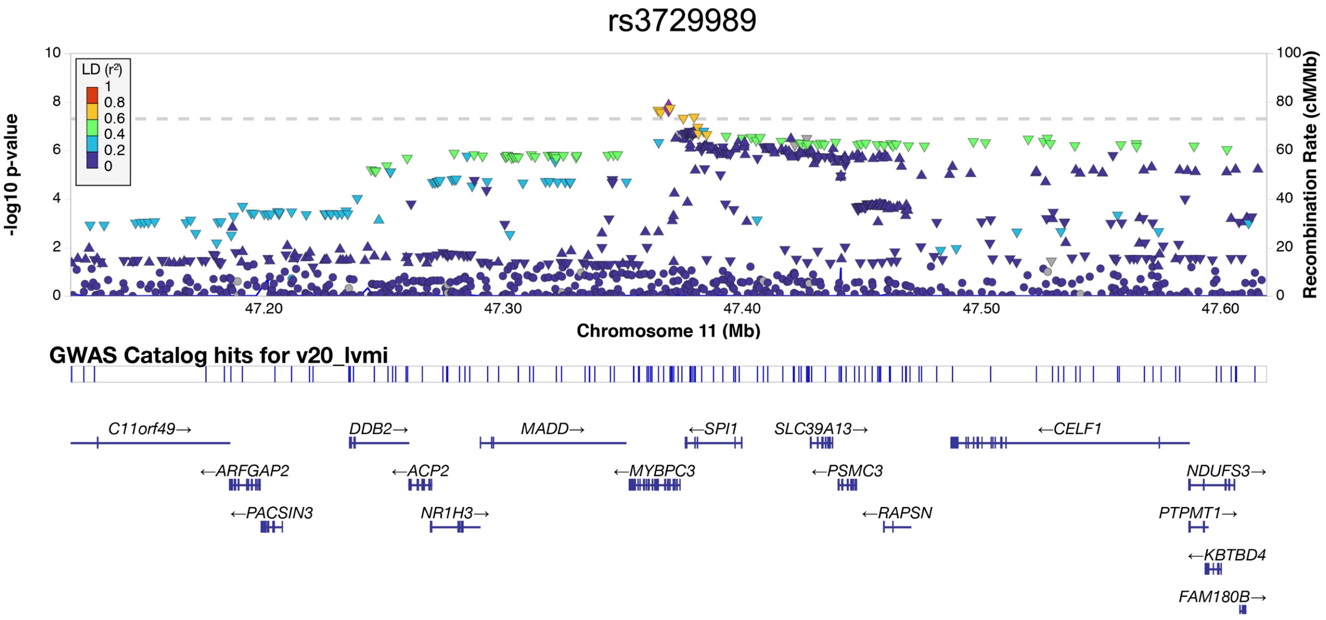
rs3729989
-log10 p-value
Recombination Rate (cM/Mb)
LD (r²)
Chromosome 11 (Mb)
GWAS Catalog hits for v20_lvmi
C11orf49→
DDB2→
MADD→
←SPI1
SLC39A13→
←CELF1
←ARFGAP2
←ACP2
←MYBPC3
←PSMC3
NDUFS3→
←PACSIN3
NR1H3→
←RAPSN
PTPMT1→
←KBTBD4
FAM180B→

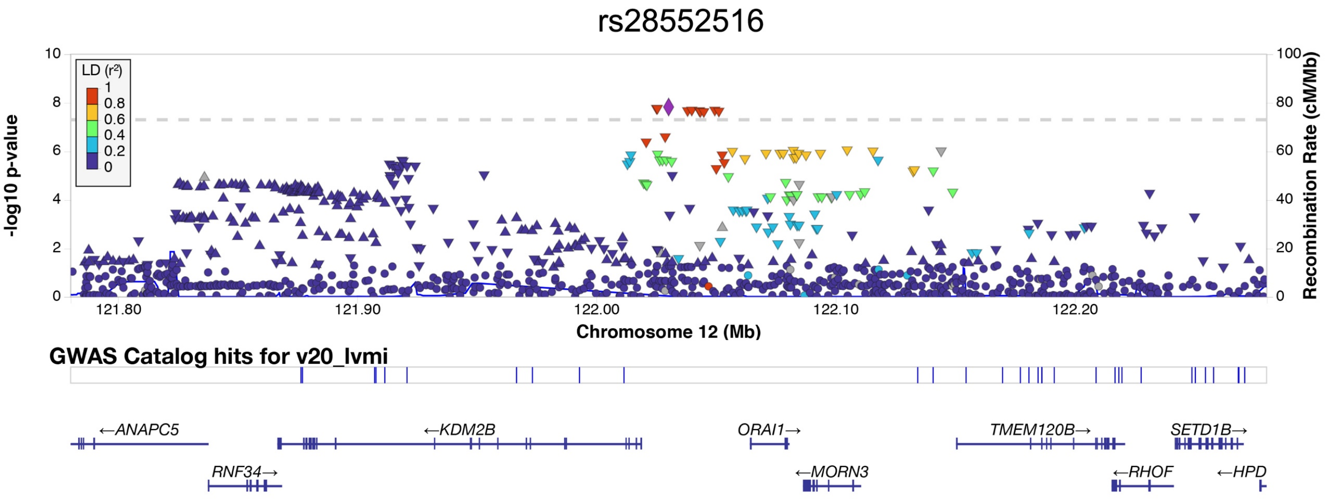
rs28552516
-log10 p-value
Recombination Rate (cM/Mb)
LD (r²)
Chromosome 12 (Mb)
GWAS Catalog hits for v20_lvmi
←ANAPC5
←KDM2B
ORAI1→
TMEM120B→
SETD1B→
RNF34→
←MORN3
←RHOF
←HPD

rs6598541

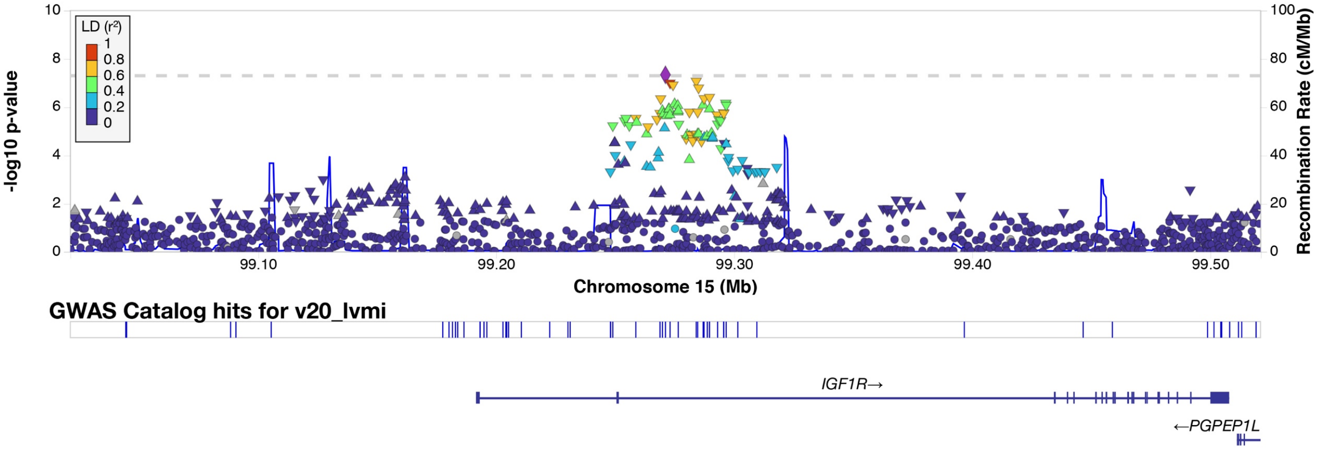

rs56252725

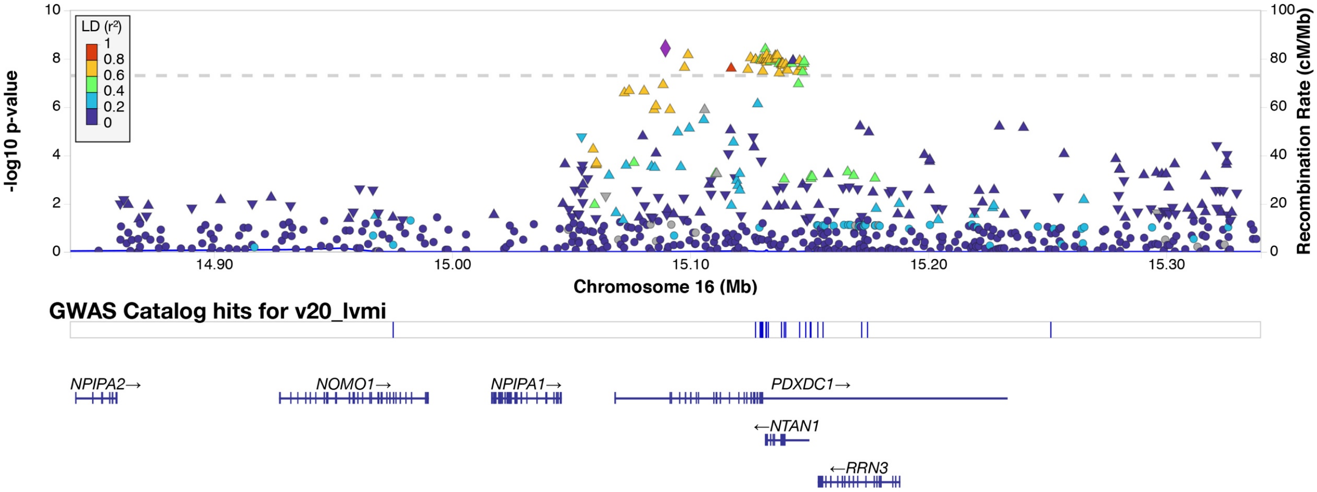

rs6503451

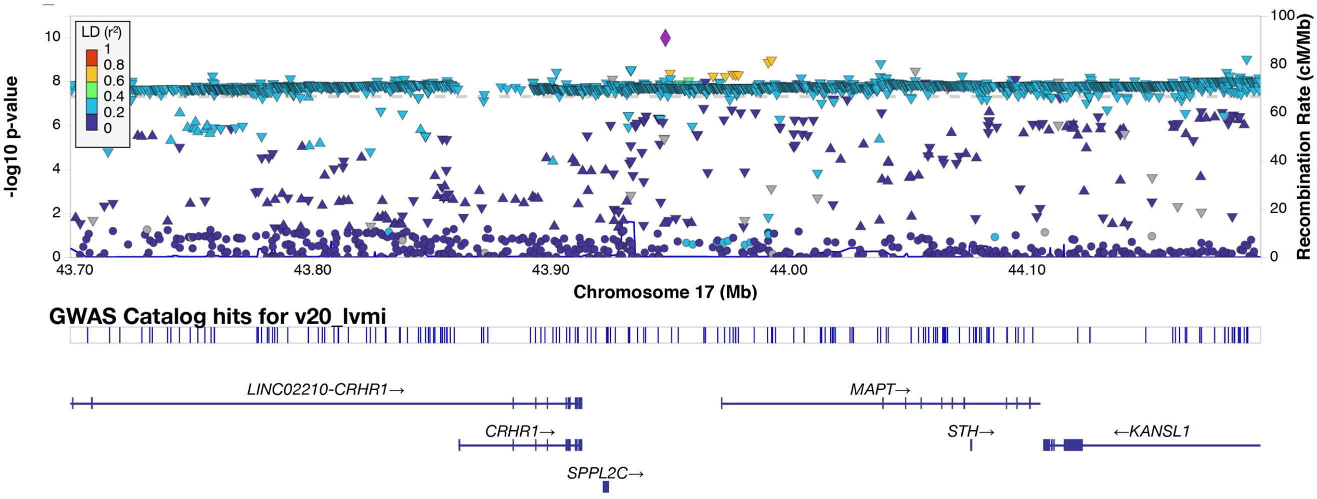

rs199501

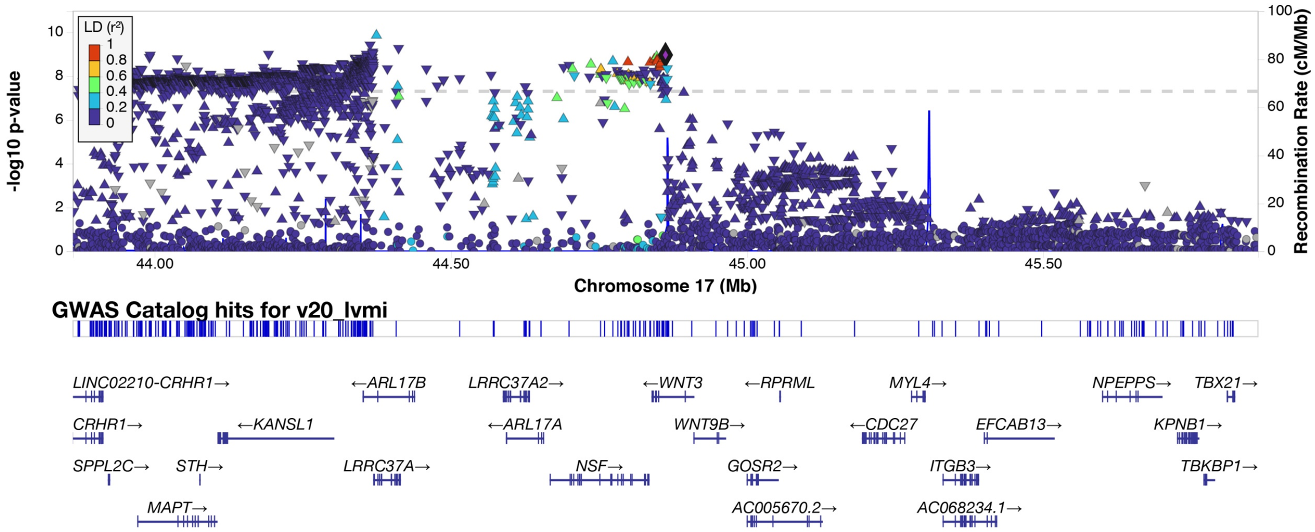

rs62621197

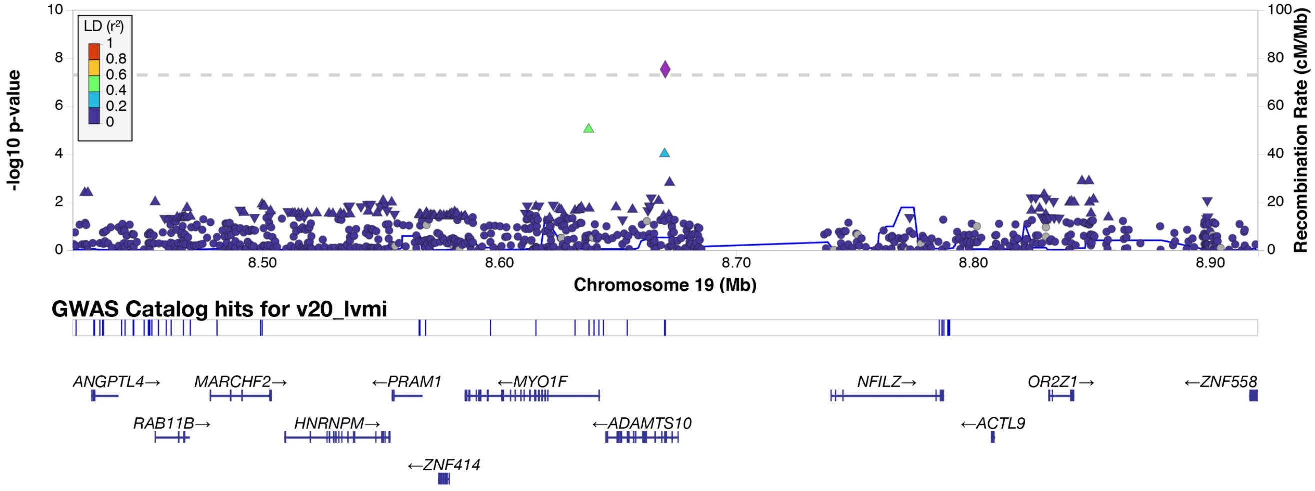

rs143973349 (EUR only)

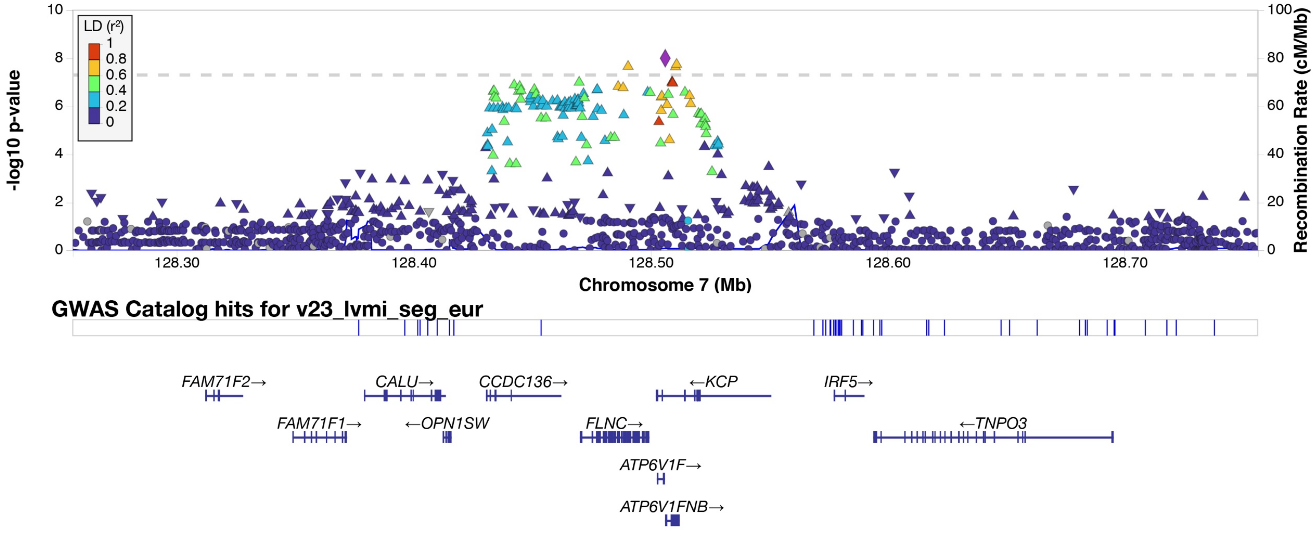

rs142032045 (EUR only)

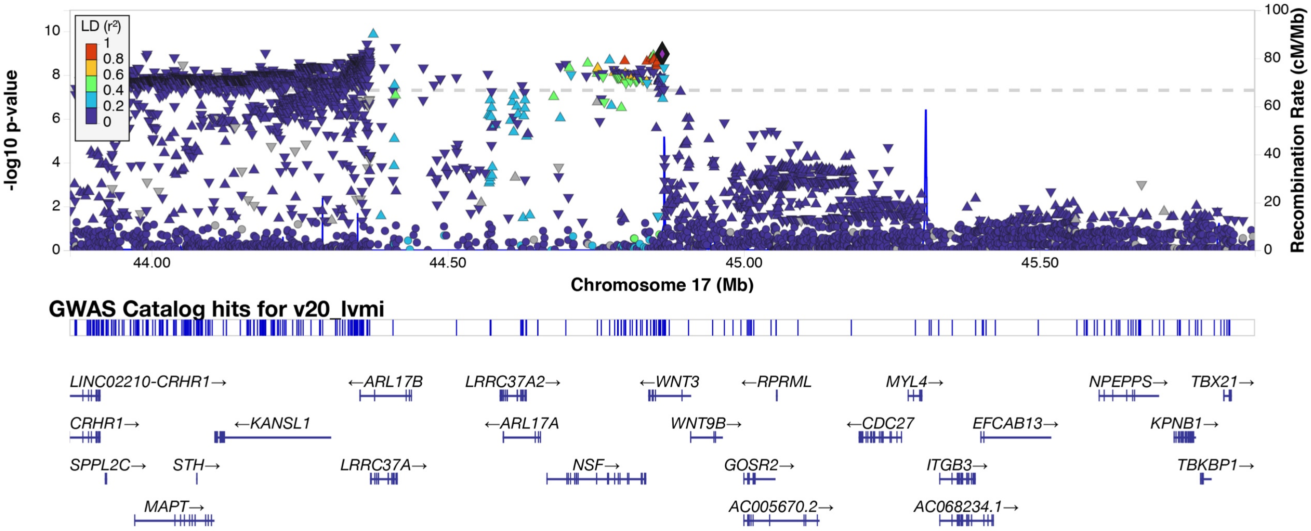

For each plot, the lead single-nucleotide polymorphism (SNP) is depicted by the purple diamond and labeled above each plot. Adjacent SNPs are depicted as circles, with the degree of linkage disequilibrium with the sentinel SNP shown by color (see legend). Linkage disequilibrium information is obtained using the All Populations 1,000G reference panel for the primary analysis variants, and the European 1,000G reference panel for the European-only variants. Genes in the region and their locations are shown at the bottom of each plot. The y-axis plots the negative $\log_{10}$ of the two-sided p-value corresponding to BOLT-LMM χ^2 statistic. The hashed horizontal line denotes genome-wide significance ($p<5x10^{-8}$).

Supplementary Figure 3. Manhattan plot of unindexed LV mass GWAS

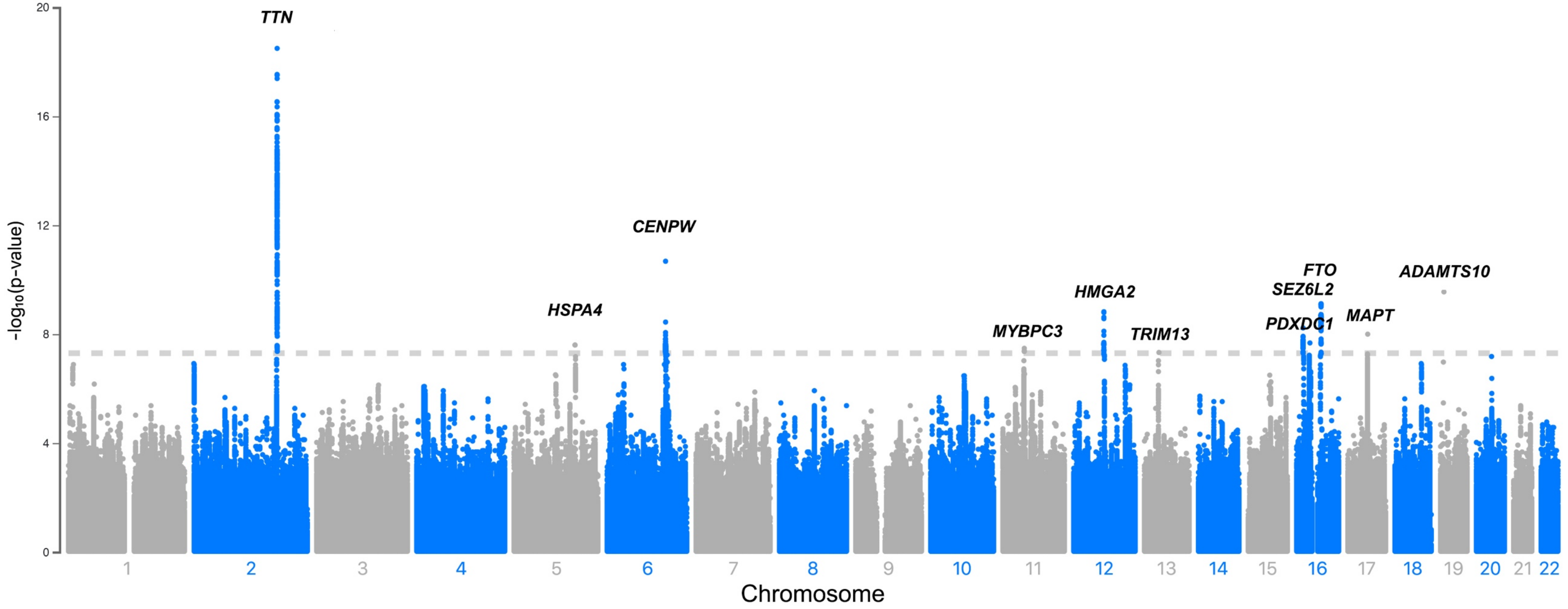

Depicted across increasing chromosome (x-axis) are the results of the European ancestry subset GWAS of left ventricular mass index. The y-axis plots the negative $\log_{10}$ of the two-sided p-value corresponding to BOLT-LMM χ^2 statistic. Variants meeting the standard multiplicity correction for genome-wide significance ($p<5x10^{-8}$, depicted by hashed horizontal line), are labeled by the closest gene to the lead variant.

Supplementary Figure 4. Quantile-quantile plot of LVMI GWAS for European-ancestry

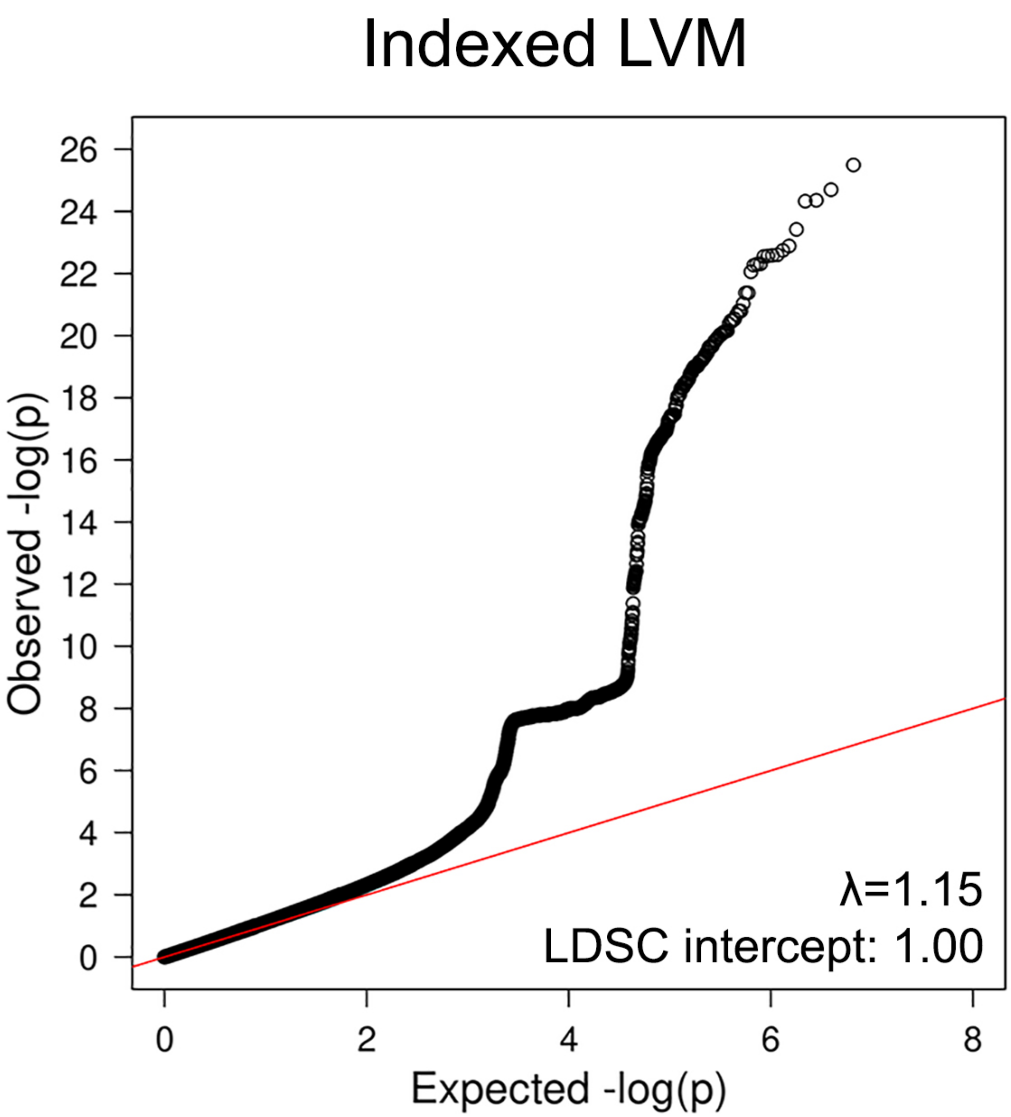

sample

Points denote two-sided p-values corresponding to the BOLT-LMM χ^2 statistic. The lambda genetic control factor was 1.15. Linkage disequilibrium score regression analysis[33] revealed an intercept of 1.00, suggesting that the elevated lambda value was attributable to polygenicity rather than inflation.

Supplementary Figure 5. Manhattan plot of European-ancestry GWAS

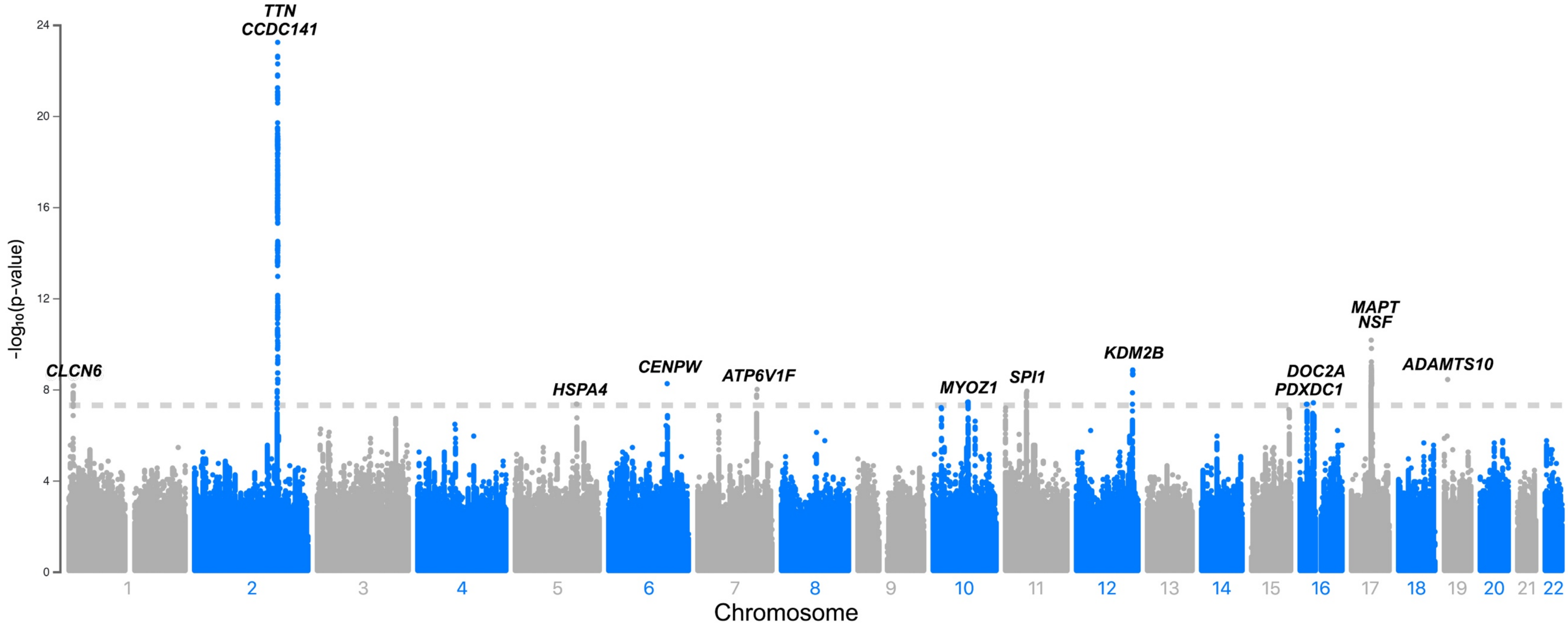

Depicted across increasing chromosome (x-axis) are the results of the European ancestry subset GWAS of left ventricular mass index. The y-axis plots the negative $\log_{10}$ of the two-sided p-value corresponding to BOLT-LMM χ^2 statistic. Variants meeting the standard multiplicity correction for genome-wide significance ($p<5x10^{-8}$, depicted by hashed horizontal line), are labeled by the closest gene to the lead variant.

Supplementary Figure 6. Two-sample Mendelian randomization plots

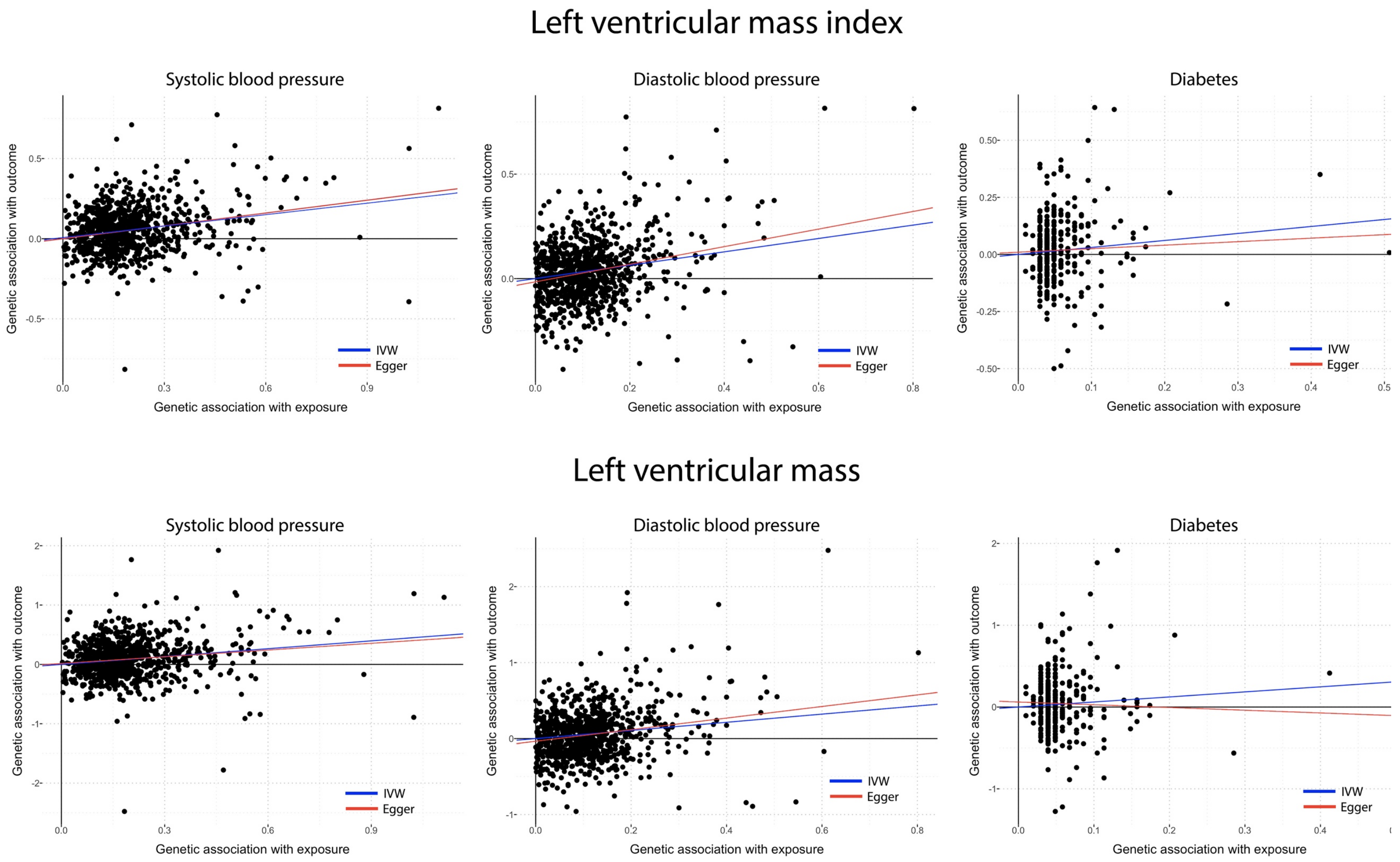

Depicted are scatterplots depicting results of two-sample Mendelian randomization. Each point is a genetic variant, the x-axis depicts strength of association between the variant and the exposure (i.e., systolic blood pressure, diastolic blood pressure, and diabetes, as labeled above each plot). The y-axis depicts strength of association between the variant and the outcome (i.e., left ventricular mass index in the top panels, and left ventricular mass in the bottom panels. Each plot depicts the result of inverse variance weighted regression (IVW, blue) and MR-Egger regression (red). A red line crossing the origin (y-intercept close to zero) suggests absence of substantial directional pleiotropy in the genetic instrument.

Supplementary Figure 7. Distribution of CMR-derived LV mass

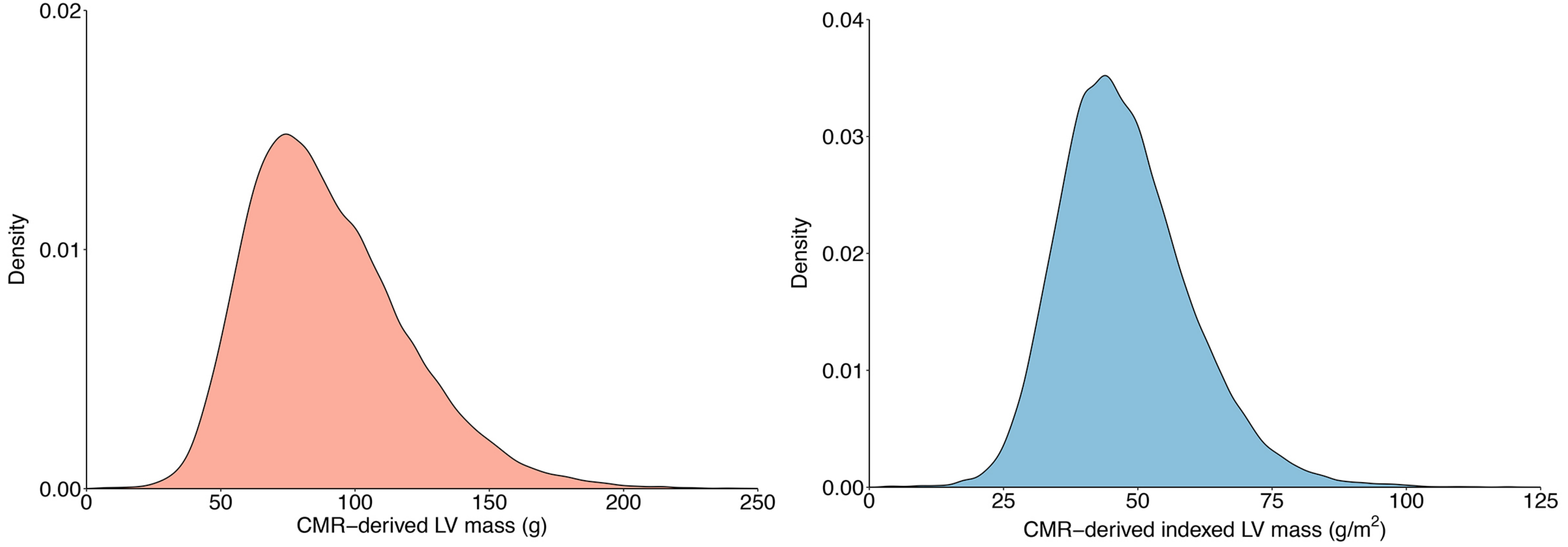

Depicted is the distribution of CMR-derived LV mass (LVM, left) and LV mass index (LVMI, right) within the UK Biobank phenotypic sample (N=44,375). The y-axis depicts the relative probability of an encountering a given value on the x-axis. For graphical purposes, four high outlying observations for LVM are not shown.

Supplementary References

1. Aung, N. *et al.* Genome-Wide Analysis of Left Ventricular Image-Derived Phenotypes Identifies Fourteen Loci Associated With Cardiac Morphogenesis and Heart Failure Development. *Circulation* **140**, 1318–1330 (2019).
2. Giri, A. *et al.* Trans-ethnic association study of blood pressure determinants in over 750,000 individuals. *Nat Genet* **51**, 51–62 (2019).
3. Del Greco M, F. *et al.* Genome-wide association analysis and fine mapping of NT-proBNP level provide novel insight into the role of the MTHFR-CLCN6-NPPA-NPPB gene cluster. *Hum Mol Genet* **20**, 1660–1671 (2011).
4. Pirruccello, J. P. *et al.* Analysis of cardiac magnetic resonance imaging in 36,000 individuals yields genetic insights into dilated cardiomyopathy. *Nat Commun* **11**, 2254 (2020).
5. Herman, D. S. *et al.* Truncations of titin causing dilated cardiomyopathy. *N Engl J Med* **366**, 619–628 (2012).
6. Satoh, M. *et al.* Structural analysis of the titin gene in hypertrophic cardiomyopathy: identification of a novel disease gene. *Biochem Biophys Res Commun* **262**, 411–417 (1999).
7. Choi, S. H. *et al.* Association Between Titin Loss-of-Function Variants and Early-Onset Atrial Fibrillation. *JAMA* **320**, 2354–2364 (2018).
8. Eppinga, R. N. *et al.* Identification of genomic loci associated with resting heart rate and shared genetic predictors with all-cause mortality. *Nat Genet* **48**, 1557–1563 (2016).
9. Hanscombe, K. B. *et al.* The genetic case for cardiorespiratory fitness as a clinical vital sign and the routine prescription of physical activity in healthcare. *Genome Med* **13**, 180 (2021).
10. Kichaev, G. *et al.* Leveraging Polygenic Functional Enrichment to Improve GWAS Power. *Am J Hum Genet* **104**, 65–75 (2019).
11. the Million Veteran Program *et al.* Genetic analysis of over 1 million people identifies 535 new loci associated with blood pressure traits. *Nat Genet* **50**, 1412–1425 (2018).
12. van der Harst, P. & Verweij, N. Identification of 64 Novel Genetic Loci Provides an Expanded View on the Genetic Architecture of Coronary Artery Disease. *Circ Res* **122**, 433–443 (2018).
13. Onengut-Gumuscu, S. *et al.* Fine mapping of type 1 diabetes susceptibility loci and evidence for colocalization of causal variants with lymphoid gene enhancers. *Nat Genet* **47**, 381–386 (2015).
14. Zhao, W. *et al.* Identification of new susceptibility loci for type 2 diabetes and shared etiological pathways with coronary heart disease. *Nat Genet* **49**, 1450–1457 (2017).
15. CHARGE-EchoGen Consortium *et al.* The genetics of blood pressure regulation and its target organs from association studies in 342,415 individuals. *Nat Genet* **48**, 1171–1184 (2016).
16. Roselli, C. *et al.* Multi-ethnic genome-wide association study for atrial fibrillation. *Nat Genet* **50**, 1225–1233 (2018).
17. Shah, S. *et al.* Genome-wide association and Mendelian randomisation analysis provide insights into the pathogenesis of heart failure. *Nat Commun* **11**, 163 (2020).

18. Young, W. J. *et al.* Genetic analyses of the electrocardiographic QT interval and its components identify additional loci and pathways. *Nat Commun* **13**, 5144 (2022).
19. Ntalla, I. *et al.* Multi-ancestry GWAS of the electrocardiographic PR interval identifies 202 loci underlying cardiac conduction. *Nat Commun* **11**, 2542 (2020).
20. Wu, Y. *et al.* Genome-wide association study of medication-use and associated disease in the UK Biobank. *Nat Commun* **10**, 1891 (2019).
21. Watkins, H. *et al.* Mutations in the cardiac myosin binding protein-C gene on chromosome 11 cause familial hypertrophic cardiomyopathy. *Nat Genet* **11**, 434–437 (1995).
22. Daehmlow, S. *et al.* Novel mutations in sarcomeric protein genes in dilated cardiomyopathy. *Biochem Biophys Res Commun* **298**, 116–120 (2002).
23. Ramírez, J. *et al.* Common Genetic Variants Modulate the Electrocardiographic Tpeak-to-Tend Interval. *Am J Hum Genet* **106**, 764–778 (2020).
24. Prins, B. P. *et al.* Exome-chip meta-analysis identifies novel loci associated with cardiac conduction, including ADAMTS6. *Genome Biol* **19**, 87 (2018).
25. Nielsen, J. B. *et al.* Biobank-driven genomic discovery yields new insight into atrial fibrillation biology. *Nat Genet* **50**, 1234–1239 (2018).
26. Koyama, S. *et al.* Population-specific and trans-ancestry genome-wide analyses identify distinct and shared genetic risk loci for coronary artery disease. *Nat Genet* **52**, 1169–1177 (2020).
27. Ramírez, J. *et al.* Thirty loci identified for heart rate response to exercise and recovery implicate autonomic nervous system. *Nat Commun* **9**, 1947 (2018).
28. Verweij, N. *et al.* The Genetic Makeup of the Electrocardiogram. *Cell Syst* **11**, 229-238.e5 (2020).
29. van der Harst, P. *et al.* 52 Genetic Loci Influencing Myocardial Mass. *J Am Coll Cardiol* **68**, 1435–1448 (2016).
30. Begay, R. L. *et al.* Filamin C Truncation Mutations Are Associated With Arrhythmogenic Dilated Cardiomyopathy and Changes in the Cell-Cell Adhesion Structures. *JACC Clin Electrophysiol* **4**, 504–514 (2018).
31. Valdés-Mas, R. *et al.* Mutations in filamin C cause a new form of familial hypertrophic cardiomyopathy. *Nat Commun* **5**, 5326 (2014).
32. Brodehl, A. *et al.* Mutations in FLNC are Associated with Familial Restrictive Cardiomyopathy. *Hum Mutat* **37**, 269–279 (2016).
33. Schizophrenia Working Group of the Psychiatric Genomics Consortium *et al.* LD Score regression distinguishes confounding from polygenicity in genome-wide association studies. *Nat Genet* **47**, 291–295 (2015).
