# Supplementary material for: Clinical and genetic associations of deep learning-derived cardiac magnetic resonance-based left ventricular mass
Source: Nat Commun. 2023 Mar 21;14:1558. doi: 10.1038/s41467-023-37173-w (PMC10030590; doi:10.1038/s41467-023-37173-w)
Supplement: Supplementary file 3 — Description of Additional Supplementary Files [file 41467_2023_37173_MOESM3_ESM.pdf]

File name: Supplementary Data 1

Description: Disease definitions
